# Conditional Control of Benzylguanine Reaction with the Self-Labeling SNAP-tag Protein

**DOI:** 10.1021/acs.bioconjchem.5c00002

**Published:** 2025-02-20

**Authors:** Steven E. Caldwell, Isabella R. Demyan, Gianna N. Falcone, Avani Parikh, Jason Lohmueller, Alexander Deiters

**Affiliations:** † Department of Chemistry, University of Pittsburgh, Pittsburgh, Pennsylvania 15260, United States; ‡ Department of Surgery, Division of Surgical Oncology, University of Pittsburgh, Pittsburgh, Pennsylvania 15213, United States; § Center for Systems Immunology, University of Pittsburgh, Pittsburgh, Pennsylvania 15213, United States

## Abstract

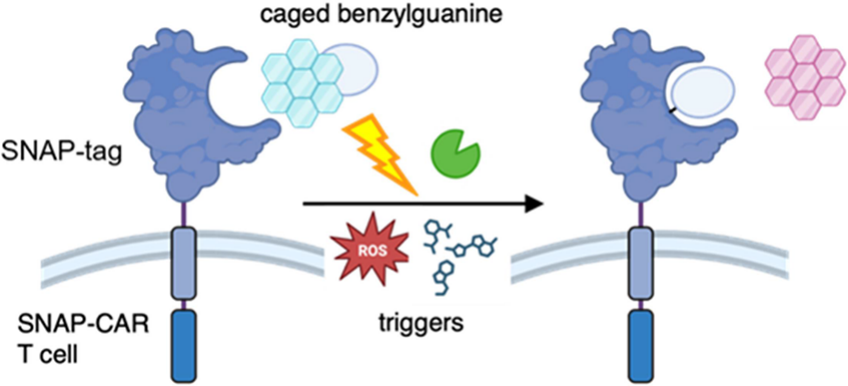

SNAP-tag, a mutant of the O^6^-alkylguanine-DNA-alkyltransferase, self-labels by reacting with benzylguanine (BG) substrates, thereby forming a thioether bond. SNAP-tag has been genetically fused to a wide range of proteins of interest in order to covalently modify them. In the context of both diagnostic and therapeutic applications, as well as use as a biological recording device, precise control in a spatial and temporal fashion over the covalent bond-forming reaction is desired to direct inputs, readouts, or therapeutic actions to specific locations, at specific time points, in cells and organisms. Here, we introduce a comprehensive suite of six caged BG molecules: one light-triggered and five others that can be activated through various chemical and biochemical stimuli, such as small molecules, transition metal catalysts, reactive oxygen species, and enzymes. These molecules are unable to react with SNAP-tag until the trigger is present, which leads to near complete SNAP-tag conjugation, as illustrated both in biochemical assays and on human cell surfaces. This approach holds promise for targeted therapeutic assembly at disease sites, offering the potential to reduce off-target effects and toxicity through precise trigger titration.

## Introduction

SNAP-tag is a self-labeling protein, derived from a mutant human O^6^-alkylguanine-DNA-alkyltransferase (hAGT), that was developed in 2003.^1^ This protein reacts with benzylguanine (BG) substrates and forms a stable thioether bond through benzyl transfer to an active site cysteine. Modification of the benzyl group with functional motifs, such as fluorophores, provides a way to selectively label proteins of interest through genetic fusion with the SNAP-tag. More recently, a version of SNAP-tag, known as SNAP*f*, was developed that has increased reactivity and has become the common protein used in the field and is the version used in this work.^2^ Moreover, various bioorthogonal reaction handles, such as an azide or a strained alkene, have been installed on the BG substrate, enabling subsequent bioconjugation reactions.^3^ SNAP-tag self-labeling has been successfully implemented in bacteria,^4^ yeast,^5^ and mammalian cells,^6,7^ as well as animals, such as mice.^8^ The labeling kinetics are fast enough to only require micromolar BG concentrations, and the labeling reaction is fully orthogonal to other reactions in biological systems.^9^ These characteristics have led to SNAP-tag being used in a variety of protein bioconjugation applications, including sensing of calcium ions,^10^ metabolites,^11^ and reactive oxygen species,^12^ protein immobilization,^13^ aggregation,^14^ and conformation elucidation,^15^ drug delivery,^16^ imaging of cellular processes,^17^ and chimeric antigen receptor (CAR) T cell reprogramming.^18^

The ability to conditionally control SNAP-tag labeling in response to different physical, chemical, or biological input stimuli has the potential to produce novel biosensors and actuators with spatially and temporally controlled activities. Since the conjugation between SNAP-tag and its substrate is irreversible, the possibility of controlling the timing of SNAP-tag labeling is a distinct advantage. This feature is useful for broader applications with SNAP-tag, as it would allow a “caged” BG attached to another substrate, specific for a certain protein of interest (POI) or cellular receptor, time to reach its target before the SNAP-tag reaction takes place.^19−24^ A chemical strategy to achieve this conditional control is to “cage” the BG, rendering it biologically inactive until a user-defined time (and location) of activation. The most prominent example of this approach is the installation of a photocaging group that undergoes photolysis upon irradiation.^19−24^ Photocaging has previously been applied to BG through modification of the N7 and N9 amines, with applications to control protein dimerization and immobilization^25^ and RNA editing through ADAR enzyme recruitment to guide RNAs.^26^ A completely different SNAP-tag ligand, chloropyrimidine, has also been optically controlled through photocaging.^27^ Furthermore, the irreversibility of SNAP-tag labeling allows for potential molecular recording, both memory of an earlier biological event and summation of the input signal over time.^28^

Here, we expand the application and utility of SNAP-tag by designing a series of caged BG compounds that can be activated with a variety of different triggers, including light, small molecules, enzymes, metal catalysts, and reactive oxygen species. This characterization includes their ability to conditionally label SNAP-CAR on primary human T cells. These decaging methods have the shared advantage of short reaction times (1 h) without the need for localized activation through tissue-specific promoters. This versatile set of compounds could be utilized to control SNAP-tag labeling in response to a variety of signals, such as the chemical makeup of the tumor microenvironment (TME), or in response to triggers that have been applied in clinical settings, such as light or small molecules,^19,29,30^ thereby holding promise to improve the utility of SNAP-tag systems for applications such as drug delivery or immunotherapy. These caging strategies are likely to be compatible with established SNAP-tag probes. Typically, functionalization of BG occurs at the benzyl group,^31^ and since our caging group is installed at the guanine N2, we expect it to be generally applicable.

## Results and Discussion

**Figure 1:**
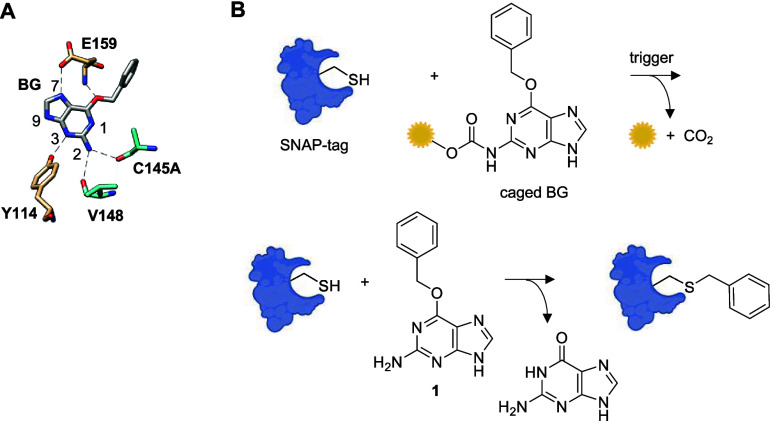
(A) Crystal structure of O^6^–BG bound to SNAP-tag-C145A with key binding interactions shown (PDB: 3KZZ). The C145A mutant does not undergo covalent labeling and was used to characterize the interactions with O6-BG.^9^ Numbers label each nitrogen for clarity. (B) Cartoon representation of conditional SNAP-tag conjugation to BG. The yellow star represents the caging group.

To provide applicability to a broad range of triggers (and the corresponding caging groups), we chose to cage the 2-NH_2_ position as it can be modified as a stable carbamate–which offers excellent leaving group qualities–is synthetically accessible, and should completely arrest SNAP-tag binding.^25^ We hypothesized that caging at this position would prevent reaction with the SNAP-tag protein through steric blocking of key interactions in the active site, such as hydrogen bonding between the 2-NH_2_ and the backbones of V148 and C145 (Figure 1A). Furthermore, the compact BG binding site will presumably be unable to accommodate a sterically demanding caging group. Thus, a caged BG will be unable to conjugate to SNAP-tag until it comes into contact with its matching trigger. Upon meeting its trigger, it decages and, following rapid decarboxylation, releases the active O^6^–BG that now undergoes an S_N_2 reaction to covalently attach the benzyl moiety to the SNAP-tag protein (Figure 1B). In this way, the SNAP-tag will only be labeled when and where the trigger is present, and the extent of labeling will depend on the amount of the trigger.

**Figure 2:**
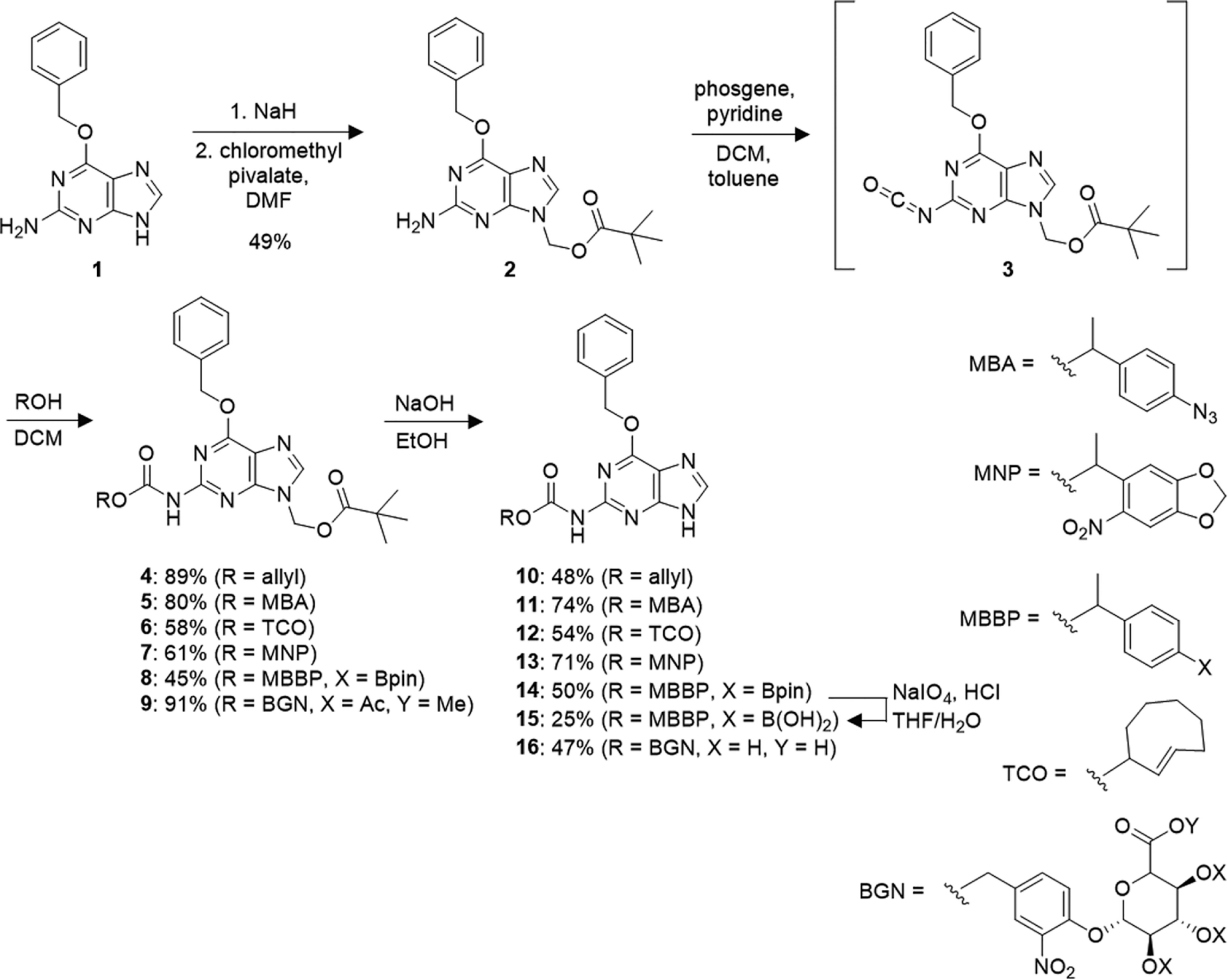
Synthetic routes to caged benzylguanines **10**–**16**.

The synthesis of the caged BGs follows a unified route that begins with the protection of the N9 in **1** with a pivaloyloxymethyl (POM) group,^32^ which we found to be necessary to avoid side reactions in subsequent steps (Figure 2). Then, isocyanate intermediate **3** is formed through treatment of **2** with phosgene, which in turn is converted into a carbamate upon the addition of the alcohol-based caging group. This step required complete consumption of the starting material **2** since its separation from the products **4**–**9** by flash column chromatography was surprisingly difficult. Attempts to form **3** through treatment with triphosgene and oxalyl chloride produced only impure or degraded material, and the direct reaction of **1** with chloroformate versions of the caging groups did not allow for the isolation of pure products. Eventually, we found that using phosgene in the presence of pyridine under a strict inert atmosphere provides a complete conversion of **1** to **3** and allows for isolation of carbamates **4**–**9** in high yields, apart from **6** and **7**, which had lower yields due to either the formation of side products or difficulty with purification of the desired products through column chromatography. A simple basic deprotection is used to remove the POM group and completes the assembly of compounds **10**–**13** and **16**. Initial work used **14** in all assays as well, but it was noted that the compound readily underwent hydrolysis to the boronic acid, which made assessing the decaging efficiency more complex. Therefore, **14** was fully hydrolyzed to the boronic acid by using sodium periodate and hydrochloric acid to yield **15**. This final step was generally high-yielding, and pure products were obtained through simple precipitation, with the exception of **10**, **12**, and **14**. Purification of these compounds via flash chromatography was difficult and may have contributed to the lower yields.

**Figure 3:**
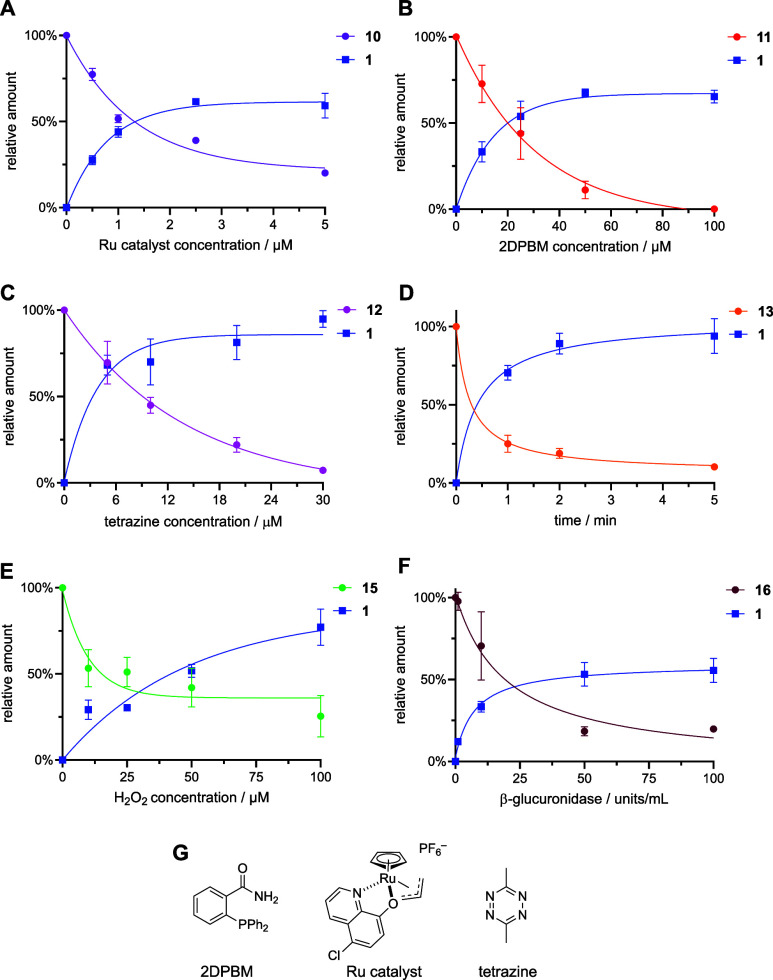
HPLC assay showing decaging of (A) **10** with a Ru catalyst, (B) **11** with 2DPBM, (C) **12** with tetrazine, (D) **13** with 365 nm light, (E) **15** with H_2_O_2_, and (F) **16** with β-glucuronidase. All caged compounds were analyzed at 20 μM concentration, reacted for 1 h, and each data point was normalized to an internal standard (20 μM *p*-nitrobenzyl alcohol). Data are averages from three replicates and error bars represent standard deviations. Graphs were generated with an exponential one phase decay model. (G) Structures of small molecule triggers.

With **10**–**16** in hand, we first analyzed the reactivity of each compound toward its matching trigger and the release of **1** by HPLC (Figure 3). Each compound was incubated in Tris buffer with 4% DMSO, and *para*-nitro benzyl alcohol was used as an internal standard. Incubations with each complementary trigger, with the exception of **13**, were carried out for 1 h at room temperature (for **10**–**12**, **15**) or 37 °C (for **16**) (Figure S3). The simple alloc-BG **10** was subjected to increasing amounts of a ruthenium(II) catalyst that has been shown to effectively cleave alloc-protected amines under physiological conditions.^33^ This transition metal-mediated allyl removal provided a 60% release of **1** in the presence of 0.25 equivalents of catalyst. While **10** does not release quantitative amounts of **1**, this result is consistent with literature reports, as it has been shown that this specific catalyst only releases about 75% of its caged material after a 2-h reaction.^33,34^ Higher amounts of decaging would likely be seen with a longer reaction time or even with the use of a more active catalyst. It is known that the ligands coordinated to the Ru catalyst can greatly affect the decaging ability, so a screen of different Ru catalysts to find the optimal one for this substrate could improve the decaging.^35^ The azido-containing BG **11** is decaged by the small molecule phosphine 2-(diphenylphosphanyl) benzamide (2DPBM), which proceeds through a Staudinger reduction of the azide with subsequent 1,6-elimination and the release of CO_2_. Here, we observed a dose-dependent formation of **1**, leveling off at 65% when treated with 2DPBM at 100 μM. Similar to **10**, **11** did not release quantitative amounts of **1**, but this is consistent with other literature reports that report incomplete decaging through this Staudinger reduction method, even when the caged species is completely consumed.^36−40^

This small molecule phosphine has previously been shown to be nontoxic to mammalian cells and aquatic embryos.^38−42^ Additionally, highly efficient activation of the TCO-caged BG **12** was observed, yielding a 90% release of **1** in the presence of 30 μM of dimethyl tetrazine via a “click-to-release” inverse electron demand Diels–Alder (IEDDA) reaction.^43−47^ The photocaged-BG **13** was found to be one of the most effective at releasing **1**, with a 90% activation within 2 min of 365 nm irradiation. The boronate-caged BG **15** shows about 75% decaging and subsequent release of native compound **1** when treated with 100 μM of H_2_O_2_, which is consistent with levels found in the TME,^48^ and applications of other boronate-based probes.^49−51^ Finally, the β-glucuronide-caged BG **16** demonstrated specific decaging in the presence of β-glucuronidase, an enzyme that is naturally overexpressed in the TME.^52^

These results demonstrate both that a significant amount of **1** can be released upon decaging and that the extent of decaging can be controlled in a dose-dependent fashion. In this way, different caging strategies can be implemented into a variety of SNAP-tag applications depending on which trigger will be the most advantageous. For example, transition metal catalysis, although limited in scope, is a reliable bioorthogonal reaction that has been shown to cleave alloc-protected fluorophores inside cells, demonstrating possible viability as a decaging trigger for cellular programming purposes.^53^ Several examples of transition metal catalysis have been implemented in a biological context, such as the Sonogashira coupling of a ubiquitin protein,^54^ a Suzuki reaction to label extracellular surfaces,^55^ and Pd-catalyzed prodrug activation in zebrafish embryos.^56^ Furthermore, the small molecule phosphine and tetrazine triggers are useful bioorthogonal tools that can be exploited for their fast activation kinetics in cellulo,^57^ in mice,^58,59^ or in zebrafish embryos.^39,42^ The bioorthogonal nature of both the azide and TCO functionalities ensures that these caging groups react only with their respective triggers, independent of the chemical environment in cells and animals. The boronate-caged BG **15** is promising in the development of targeted therapeutics, as it offers decaging by reactive oxygen species, specifically H_2_O_2_ which is found in the TME,^60^ under hypoxic conditions,^61^ and in inflamed tissue.^62^ Furthermore, the glucuronide-caged BG **16** demonstrates the ability of an enzyme, β-glucuronidase, to trigger the release of **1**, albeit with only a 50% recovery. We first conducted the assay at pH 7.4, with 15 min incubation times, which did not give a dose–response but instead showed a plateau of **1** after 10 units/mL. With a longer incubation time and adjusting to the optimal pH of 6.8, we see an increase in the dose–response of both consumption of **16** and release of **1**. Enzyme efficiency has been reported to be substrate specific and β-glucuronidases from different organisms show different yields for different substrates, meaning that the caged molecule attached to the glucuronic acid, here **1**, affects enzyme hydrolysis.^63^ With this result in mind, we attempted the same decaging assay with β-glucuronidase from *P. vulgata* (data not shown), as this was reported to decage more efficiently for different substrates.^63^ However, in our hands, we found that the enzyme from *E. coli* remained the most effective β-glucuronidase to decage **16**. Nonetheless, the enzyme trigger is promising, as elevated β-glucuronidase activity has been reported in a wide range of malignancies.^52,64,65^

**Figure 4:**
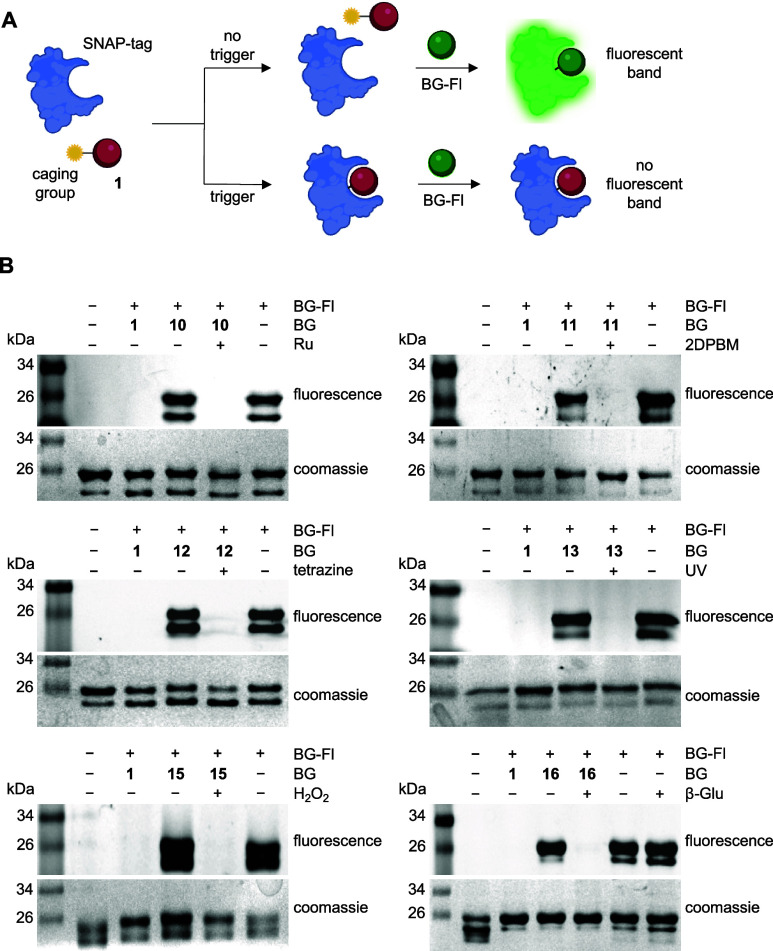
(A) Cartoon representation of the gel-based pulse chase experiment to test conditional reaction of BG with SNAP-tag. The red sphere represents compound **1** and the green sphere represents BG-Fl. (B) Gel results for the caged compounds (20 μM) conjugation to SNAP-tag (5 μM) in the presence and absence of their respective triggers. Each incubation is 2 h long and is carried out at 37 °C. SNAP-tag protein sometimes appears as a double band, as documented in the literature.^4^

After decaging of **10**–**16** was shown to generate **1** upon exposure to each respective trigger, we next sought to validate conditional conjugation to the recombinant SNAP-tag protein. To this end, we developed a gel-based pulse chase experiment (Figure 4A). In this assay, an initial incubation of the caged compound and recombinantly expressed SNAP-tag protein was performed in either the presence or the absence of the respective trigger. We expected the sample that contains the trigger to decage and release **1** which would subsequently bind to SNAP-tag in a covalent fashion, while the nonactivated and caged benzylguanine would be unable to react due to the presence of the caging group. Next, BG-fluorescein (BG-Fl) was added and, in the case of successful, trigger-initiated release of **1**, would be unable to bind to SNAP-tag (due to the already bound **1**). However, in the absence of the trigger, BG-Fl itself would react with SNAP-tag and covalently install the fluorescent moiety onto the protein. Then each sample was analyzed by SDS-PAGE and fluorescence imaging to determine the presence or absence of a fluorescent SNAP-tag band. Even though it is likely that **1** has different labeling kinetics than BG-Fl,^9^ the generous incubation times and excess of the labeling substrate likely renders this difference insignificant. The excess of the caged compound, since it is completely inactive, also ensures complete conditional control over protein labeling, as qualitatively visualized. While decaging efficiency cannot be assessed in these protein labeling experiments, the aforementioned HPLC assays provide this information.

When this assay was applied to all six caged-BGs, each compound showed a prominent fluorescent band in the nontreated experiments (Figure 4B). These results validate our hypotheses that caging at the exocyclic amine very effectively blocks the active site of SNAP-tag and that **10**–**16** does not prematurely decage or degrade. Moreover, there is a near complete lack of a fluorescent band in the trigger-treated samples, suggesting that the amount of **1** released is significant enough to restore complete conjugation to the SNAP-tag and blocking of its active site.

Taken together, these results indicate that complete conditional control of the covalent reaction between a benzylguanine and the SNAP-tag protein can be obtained by using six different caging strategies and that efficient conditional control is achieved with six different triggers. Furthermore, it demonstrates that even with nonquantitative decaging, complete conditional control over SNAP-tag conjugation can be reached. The implementation of these caging techniques expands the temporal and spatial precision of SNAP-tag applications.

**Figure 5:**
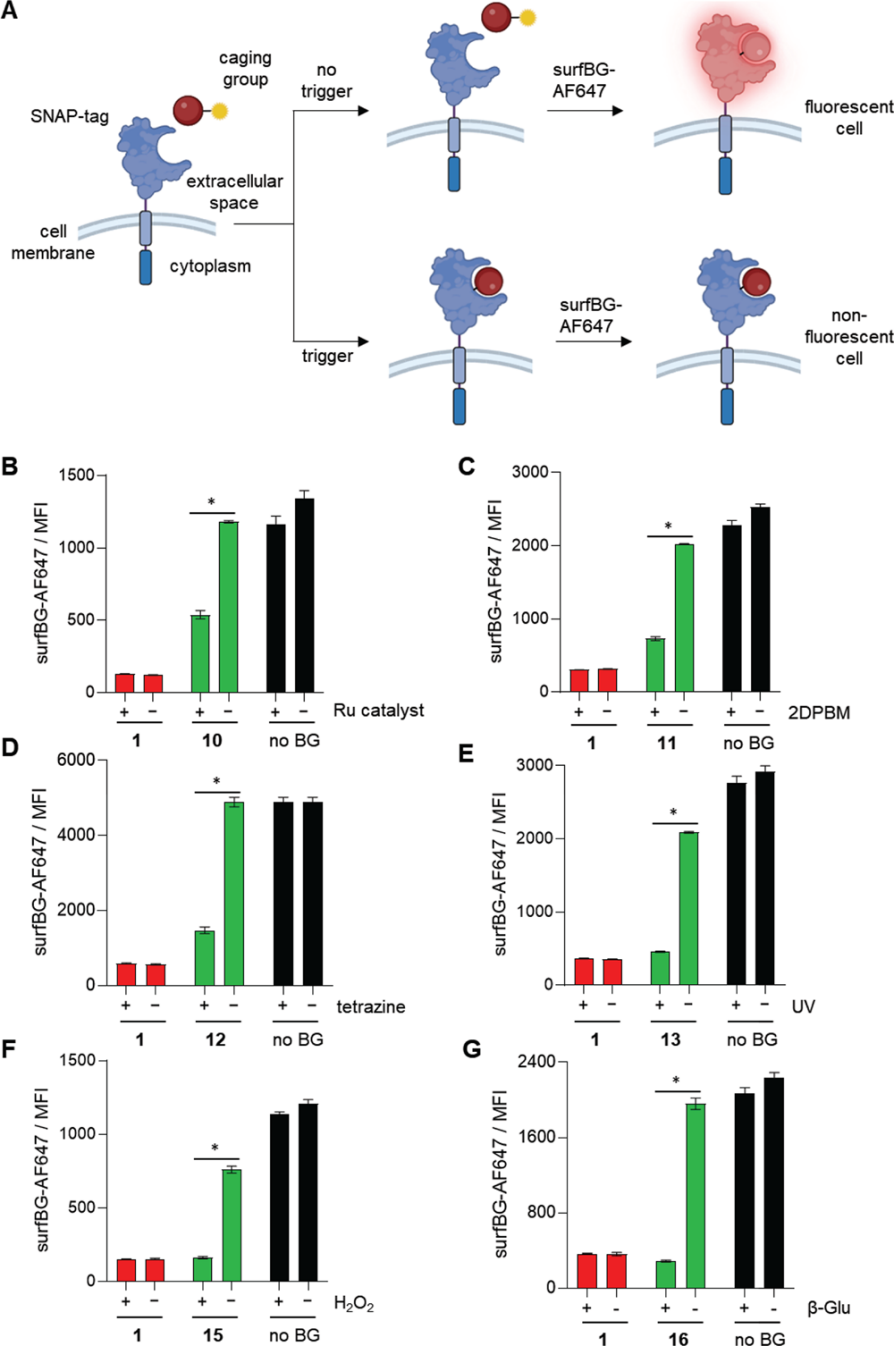
(A) Cartoon representation of the flow cytometry-based pulse chase experiment to test conditional labeling of SNAP-CAR T cells with the caged BG compounds, incubated with each caged BG for 3 h at 37 °C. (B) Mean fluorescence intensity (MFI) of surfBG-AF647 dye staining of primary human SNAP-CAR T cells treated with **10** in the presence or absence of the Ru catalyst, (C) treated with **11** in the presence or absence of 2DPBM, (D) treated with **12** in the presence or absence of tetrazine, (E) treated with **13** in the presence or absence of UV light, (F) treated with **15** in the presence or absence of H_2_O_2_, and (G) treated with **16** in the presence or absence of β-glucuronidase. Three biological replicates were performed, and error bars denote standard deviations. Parametric unpaired *t* tests were performed with the Holm-Šídák correction and no assumption about consistent SDs to compare trigger positive and negative trigger conditions. “*” denotes a significance of *p* < 0.01.

To further demonstrate the utility and relevance of the caged BGs to the control of biological systems, we tested whether we could conditionally label human cells expressing SNAP-tag receptors in the presence or absence of different trigger conditions (Figure 5). We first generated primary human T cells expressing a SNAP-tag chimeric antigen receptor (CAR) by transducing them with a gamma retroviral expression vector (Figure S1).^18^ We then incubated the SNAP-tag-CAR T cells with each caged BG in the presence or absence of its cognate trigger stimulus for 2 h (note that the light trigger was applied for 30 s). Following this incubation, cells were washed and then treated with a SNAP-tag surface-staining fluorescent dye (surfBG-AF647) for 1 h (Figure S2). This experiment was expected to lead only to surfBG-AF647 staining of T cells that were not previously labeled with the decaged BG (Figure 5A). A minor reduction in staining was observed in the absence of a trigger, potentially indicating some premature decaging within the more complex cellular environment. In agreement with our gel-based results, we found that the caged BGs could be efficiently and specifically decaged by their cognate triggers, leading to a reaction with cell surface SNAP-tag (Figure 5B−G). For **13**, **15**, and **16**, the staining was blocked at comparable levels to the noncaged BG control **1** (Figure 5E–G). While **10**, **11**, and **12** showed incomplete blocking, a greater than two-fold change was observed for each trigger. In developing caged molecules for future control of receptor functions, experiments will be required to determine if this dynamic range is sufficient for differential activity in ON versus OFF states. If incomplete decaging is the issue, then the dynamic range could be improved with a higher dose of the caged molecule.^66^

## Conclusions

SNAP-tag is a versatile self-labeling enzyme that has been used in a wide range of protein bioconjugation applications. Examples include fluorescent tagging of proteins for live cell imaging and super-resolution imaging,^67^ delivery of toxic drug payloads,^68^ antibody targeting of universal CAR T cells,^18^ and antibody targeting of viruses and virus-like particles for cell-specific biomolecule delivery.^70^ While the SNAP-tag protein’s ability to conjugate to its benzylguanine substrate has been placed under optical control through photocaging, other triggers for the conditional activation of the bioconjugation reaction have not been implemented. In this study, we presented six caged BG compounds, one that follows a traditional light-activation approach and five other compounds that decage through different chemical and biochemical stimuli, such as small molecules, transition metal catalysts, reactive oxygen species, and enzymes.

Each of the caged compounds was shown to release significant amounts of native O^6^–BG when exposed to their respective triggers. Moreover, the installation of the caging groups completely prevented binding to SNAP-tag in biochemical protein labeling assays without significant background decaging or decomposition. Furthermore, when treated with nontoxic amounts of exogenous triggers^39,71^ or amounts of endogenous triggers that resemble concentrations found in the TME,^72−74^ a complete return of SNAP-tag conjugation was observed. While some compounds do not quantitatively decage in the presence of their triggers, full SNAP-tag conjugation is achieved through an increase in the number of equivalents of the caged molecule. This is possible because they display complete inactivity when left untreated, thus increasing the equivalents does not lead to background labeling.^66^ This result and demonstration of conditional labeling of SNAP-CAR T cells sets the stage for future potential advancements in CAR T cell therapy. One could imagine these caged compounds conjugated to antigen-specific antibodies, implementing our conditional SNAP-tag conjugation into already established treatments. Future research into improving spatiotemporal control and dose-titration optimization might alleviate off-target effects and undesired toxicity in patients.

Conditional control over SNAP-tag labeling with caged BGs enables the molecular recording of transient chemical stimuli. In contrast to biosensors that directly read out signals in the presence of stimuli, such as sensors based on circular permuted fluorescent proteins,^75^ SNAP-tag irreversibly binds to BG leading to SNAP-tag labeling that persists for the lifetime of the SNAP-tag protein. Given the stability of our caged BGs, the system will allow for sensing the corresponding physical, chemical, and biological triggers and perform integration of signals over time, such as multiple light pulses or exposures to ROS, thereby potentially providing a more informative picture of biochemical history than individual snapshots. While recorders based on DNA editing are suitable for long-term storage, such as multiple generations of bacterial growth,^76^ covalent protein labeling may be more suitable for memory over shorter time scales^28^ and with a potential output of biochemical and therapeutic activity.^18^

## Methods

### Gel-Based Pulse-Chase Experiment of **10**–**12**, **15**

The SNAP-tag protein (3.1 μL, 5 μM) was incubated in Tris buffer (20 μL, 50 mM Tris-HCl, 250 mM NaCl, 0.1 mM EDTA, pH 7.4) with **10**–**13** and **15** (2.0 μL, 20 μM) and its specific trigger (2.0 μL, 100 μM) for 2 h at 37 °C. After this initial conjugation, BG-Fl (2.0 μL, 20 μM) was added, and the samples were incubated again for 2 h at 37 °C. Then, a 6× Laemmli loading buffer was added to each sample, which was denatured at 90 °C for 5 min. Each sample (10 μL) was then loaded and resolved on a 12% SDS-PAGE stacking gel for 20 min at 60 V, followed by 60 min at 140 V. The gel was imaged with UV Trans Illumination with a 605/50 Filter and then Coomassie-stained; all images were taken with a BioRad ChemiDoc Universal Hood III system and processed using Image Lab, ImageJ v6.1.

### Gel-Based Pulse-Chase Experiment of **13**

The SNAP-tag protein (3.1 μL, 5 μM) was incubated in Tris buffer (20 μL, 50 mM Tris-HCl, 250 mM NaCl, 0.1 mM EDTA, pH 7.4) with **13** (2.0 μL, 20 μM), and the samples were irradiated with a 365 nm LED (Mouser Electronics single color Luxigen LZ1MCPCB, 66 mW/cm^2^) for 2 min. Light intensity measurements were performed using a ThorLabs power and energy meter console (PM200) with a sensor (S170C). Following irradiation, the samples were incubated for 2 h at 37 °C. After this initial conjugation, BG-Fl (2.0 μL, 20 μM) was added and the samples were incubated for 2 h at 37 °C. Then 6× Laemmli loading buffer was added to each sample, followed by heating at 90 °C for 5 min. Each sample (10 μL) was then loaded and resolved on a 12% SDS-PAGE stacking gel for 20 min at 60 V, followed by 60 min at 140 V. The gel was imaged with UV Trans Illumination with a 605/50 filter and then Coomassie stained; all images were taken with a BioRad ChemiDoc Universal Hood III system and processed using ImageJ v6.1.

### Gel-Based Pulse-Chase Experiment of **16**

Beta-glucuronidase protein (2.0 μL, 1000 units/mL, purchased from Sigma-Aldrich, G7396) was incubated in Tris buffer (50 mM Tris-HCl, 250 mM NaCl, 0.1 mM EDTA, pH 7.4) with **15** (2.0 μL, 20 μM) for 4 h at 37 °C. After this initial incubation, the enzyme was denatured at 90 °C for 5 min. SNAP-tag protein (2.0 μL, 5 μM) was added in Tris buffer and allowed to incubate for 2 h at 37 °C, followed by subsequent incubation with BG-Fl (2.0 μL, 20 μM), again for 2 h at 37 °C. Then 6× Laemmli loading buffer was added to each sample, which was denatured at 90 °C for 5 min. Each sample (10 μL) was then loaded and resolved on a 12% SDS-PAGE stacking gel for 20 min at 60 V, followed by 60 min at 140 V. The gel was imaged with UV Trans Illumination with a 605/50 Filter and then coomassie stained; all images were taken with a BioRad ChemiDoc MP and processed using Image Lab, ImageJ 6.1.

### HPLC Decaging Studies of **10**–**12**, **14**–**15**

Compounds **10**–**12**, **14** (4.0 μL, 20 μM) and **15** (4.0 μL, 200 μM) and 4-nitrobenzyl alcohol (internal standard, 4.0 μL, 20 μM) were pipetted into Tris buffer (100 μL, 50 mM Tris-HCl, 250 mM NaCl, 0.1 mM EDTA, pH 7.4) with 4% DMSO. Different concentrations of the trigger (2.0 μL) were added, and the samples were incubated at room temperature for 1 h. At the end of the hourlong incubation, each sample was immediately injected onto a Shimadzu HPLC where it was analyzed via an Agilent Zorbax SB-C18 column (5 μM, 4.6 mm × 150 mm, P/N 883975-902) with a 10 min gradient of 25–95% acetonitrile (0.1% TFA) in water (0.1% TFA). The percentage of caged BG remaining was determined by dividing the area of its corresponding peak by the area of its peak at *t* = 0, normalized to the internal standard. The percentage of **1** released was determined by dividing the area of its corresponding peak by the area of its peak from a standard solution of **1** (20 μM) in Tris buffer, normalized to the internal standard. 4-Nitrobenzyl alcohol was used as a standard because it is soluble in the buffer system, shows good absorbance, is nonreactive, and does not overlap with any other peaks in the HPLC chromatograms.

### HPLC Analysis of the Decaging of **13**

Compound **13** (4.0 μL, 20 μM) and 4-nitrobenzyl alcohol (internal standard, 4.0 μL, 20 μM) were pipetted into Tris buffer (100 μL, 50 mM Tris-HCl, 250 mM NaCl, 0.1 mM EDTA, pH 7.4) with 4% DMSO. The sample was irradiated with a 365 nm LED (Mouser Electronics single color Luxigen LZ1MCPCB, 66 mW/cm^2^) for increasing durations (0–5 min). Light intensity measurements were performed using a ThorLabs power and energy meter console (PM200) with a sensor (S170C). Each sample was then immediately injected onto a Shimadzu HPLC where it was analyzed via an Agilent Zorbax SB-C18 column (5 μM, 4.6 × 150 mm, P/N 883975-902) with a 10 min gradient of 25–95% acetonitrile (0.1% TFA) in water (0.1% TFA). The percentage of caged BG remaining was determined by dividing the area of its corresponding peak by the area of its peak at *t* = 0, normalized to the internal standard. The percentage of **1** released was determined by dividing the area of its corresponding peak by the area of its peak from a standard solution of **1** (20 μM) in Tris buffer, normalized to the internal standard. 4-Nitrobenzyl alcohol was used as a standard because it is soluble in the buffer system, shows good absorbance, is nonreactive, and does not overlap any other peaks in the HPLC chromatograms.

### HPLC Decaging Study of **16**

Compound **16** (4.0 μL, 20 μM) and 4-nitrobenzyl alcohol (internal standard, 4.0 μL, 20 μM) were pipetted into Tris buffer (100 μL, 50 mM Tris-HCl, 250 mM NaCl, 0.1 mM EDTA, pH 6.8) with 4% DMSO. Different concentrations of the trigger (2.0 μL) were added in aliquots (2.0 μL, *t* = 1, 20, and 40 min) and the samples were incubated at 37 °C for 1 h. At the end of the incubation, each sample was quenched with 0.1 M TEAA in acetonitrile (100 μL) and centrifuged for 10 min at 3000 rpm. The samples were then evaporated to dryness and redissolved in Tris buffer (100 μL, pH 6.8). Then the samples were injected onto a Shimadzu HPLC where it was analyzed via an Agilent Zorbax SB-C18 column (5 μM, 4.6 mm × 150 mm, P/N 883975-902) with a 10 min gradient of 30–60% acetonitrile (0.1% TFA) in water (0.1% TFA). The percentage of caged BG remaining was determined by dividing the area of its corresponding peak by the area of its peak at *t* = 0, normalized to the internal standard. The percentage of **1** released was determined by dividing the area of its corresponding peak by the area of its peak from a standard solution of **1** (20 μM) in Tris buffer, normalized to the internal standard.

### Cell-Based Pulse-Chase Experiments

Fifty thousand SNAP-CAR T cells were coincubated in phenol red-free RPMI with 10% FBS with the indicated amount of caged BG and respective trigger as indicated in the caption for Figure 4 in a 96-well U-bottom plate for 3 h at 37 °C in a cell culture incubator. Postincubation, cells were washed twice with FACS buffer (PBS with 2% FBS and 1% penicillin-streptomycin) spinning at 1400 rpm for 2 min. Cells were then stained with 1 μM SNAP surface dye-AF647 (Cat# S9136S, New England Biolabs) for 1 h at 37 °C in a cell culture incubator. Cells were washed twice with FACS buffer to remove the unbound dye and stained with antihuman LNGFR-BV421 antibody (Cat# 562662, BD Biosciences) at a 1:100X dilution for 30 min on ice. Cells were then again washed twice with FACS buffer to remove unbound antibodies. Finally, cells were resuspended in the FACS buffer and analyzed on an LSR Fortessa cytometer (BD Biosciences) running BD FacsDiva v9.0 software (BD Biosciences). Flow cytometry data were analyzed using a Flowjo v10.10.1 (FlowJo, LLC) software.

## Supporting Information Available

Additional experimental methods and supporting figures, gene construct for generating SNAP-CAR T cells, gating strategy for flow cytometry experiments, and spectra for new compounds (PDF)
