# Supplementary material for: Conditional Control of Benzylguanine Reaction with the Self-Labeling SNAP-tag Protein
Source: Bioconjug Chem. 2025 Feb 20;36(3):540–8. doi: 10.1021/acs.bioconjchem.5c00002 (PMC11926790; doi:10.1021/acs.bioconjchem.5c00002)
Supplement: Supplementary file 1 — bc5c00002_si_001.pdf [file bc5c00002_si_001.pdf]

Supporting Information

Conditional Control of Benzylguanine Reaction with the Self-Labeling SNAP-tag Protein

Steven E. Caldwell[1], Isabella R. Demyan[1], Gianna N. Falcone[1], Avani Parikh[2], Jason Lohmueller[2,3]* and Alexander Deiters[1,3]*

[1] *Department of Chemistry, University of Pittsburgh, Pittsburgh, PA 15260, United State*s

[2] *Division of Surgical Oncology, Department of Surgery, University of Pittsburgh, Pittsburgh PA 15213, United States*

[3] *Center for Systems Immunology, University of Pittsburgh, Pittsburgh PA 15213, United States*

Synthetic Protocols

All chemicals obtained from commercial sources Acros, Ambeed, Alfa Aesar, Fisher, Oakwood, Sigma Aldrich, and TCI Chemicals were used without purification. ^{1}H and ^{13}C NMR spectra were obtained from a Bruker Avance III 400 MHz or 500 MHz spectrometer with chemical shifts reported relative to residual $CDCl_3$ (7.26 ppm) or d_6-DMSO (2.50 ppm). HRMS was performed on a Q-Exactive (Thermo Scientific) mass spectrometer by University of Pittsburgh facilities. Note: Phosgene is hazardous and should be used with care. If possible, use of alternative, less reactive reagents such as triphosgene is encouraged.

(2-Amino-6-(benzyloxy)-9H-purin-9-yl)methyl pivalate (2). 6-(benzyloxy)-9H-purin-2-amine (500 mg, 2.07 mmol) was dissolved in dry DMF (10 mL) under argon. The solution was cooled to 0 °C and NaH (108 mg, 2.69 mmol, 60% in mineral oil, 1.3 eq) was added portion-wise. The solution was stirred at 0 °C for 30 minutes before it was warmed to room temperature. Then chloromethyl pivalate (596 uL, 4.14 mmol, 2.0 eq) was added dropwise. Upon complete addition the reaction stirred at room temperature for 1 hour, after which it was slowly neutralized with 1 M HCl. The reaction mixture was then diluted with water (30 mL) and extracted with DCM (3 x 50 mL). The resulting organic layers were washed with brine, dried over anhydrous Na_2SO_4, and concentrated. The resulting crude material was purified by flash column chromatography on SiO_2 (60% hexanes in EtOAc) to yield the desired product **2** (358 mg, 49%) as a white solid. All characterization matches literature reported data.[1]

(2-(((Allyloxy)carbonyl)amino)-6-(benzyloxy)-9H-purin-9-yl)methyl pivalate (4). Compound **2** (50 mg, 0.14 mmol) was dissolved in anhydrous DCM (3.2 mL) and anhydrous pyridine (0.140 mL) under argon. This solution was cooled to 0 °C and phosgene (0.200 mL, 0.28 mmol, 2.0 eq, 15% in toluene) was added dropwise under stirring, after which the reaction was left at 0 °C and slowly warmed to room temperature overnight. Then allyl alcohol (11.6 µL, 0.17 mmol, 1.2 eq)

was added to the reaction where it stirred at room temperature for an additional 2 hours. The volatiles were removed *in vacuo* and the resulting crude solid was purified by flash column chromatography on SiO_2 (60% hexanes in EtOAc) to yield **4** (55 mg, 89%) as an off-white solid. ^{1}H NMR (500 MHz, d_6-DMSO) δ 10.52 (s, 1 H), 8.28 (s, 1 H), 7.58 (d, 2 H, J = 6.9 Hz), 7.38 (m, 3 H), 6.07 (s, 2 H), 5.98 (ddt, 1 H, J = 17.2 10.5, 5.2 Hz), 5.62 (s, 2 H), 5.42 (dd, 1 H, J = 17.2, 1.7 Hz), 5.24 (dd, 1 H, J = 10.5, 1.7 Hz), 4.64 (d, 2 H, J = 5.2 Hz), 1.09 (s, 9 H). ^{13}C NMR (125 MHz, d_6-DMSO) δ 177.4, 160.2, 153.7, 153.1, 152.2, 143.5, 136.6, 133.6, 129.5, 128.9, 128.8, 117.8, 117.0, 68.3, 65.9, 65.2, 38.7, 27.0. HRMS $(M+H)^+$ calculated for $C_{22}H_{26}O_5N_5^+$ $(M+H)^+$ 440.1929, found 440.1939.

(2-((((4-Azidobenzyl)oxy)carbonyl)amino)-6-(benzyloxy)-9H-purin-9-yl)methyl pivalate (5). Compound **2** (50 mg, 0.14 mmol) was dissolved in anhydrous DCM (3.2 mL) and anhydrous pyridine (0.140 mL) under argon. This solution was cooled to 0 °C and phosgene (0.200 mL, 0.28 mmol, 2.0 eq, 15% in toluene) was added dropwise under stirring, after which the reaction was left at 0 °C and slowly warmed to room temperature overnight. Then a solution of the caging group (27 mg, 0.17 mmol, 1.2 eq) in anhydrous DCM (1.0 mL) was added to the reaction where it stirred at room temperature for an additional 2 hours. The volatiles were removed *in vacuo* and the resulting crude solid was purified by flash column chromatography on SiO_2 (60% hexanes in EtOAc) to yield **5** (61 mg, 80%) as a white solid. ^{1}H NMR (500 MHz, d_6-DMSO) δ 10.50 (s, 1 H), 8.28 (s, 1 H), 7.56 (d, 2 H, J = 6.6 Hz), 7.52 (d, 2 H, J = 8.5 Hz), 7.39 (m, 3 H), 7.14 (d, 2 H, J = 8.5 Hz), 6.07 (s, 2 H), 5.86 (q, 1 H, J = 13.0, 6.6 Hz), 5.59 (s, 2 H), 1.53 (d, 3 H, J = 6.5 Hz), 1.09 (s, 9 H). ^{13}C NMR (125 MHZ, d_6-DMSO) δ 177.4, 160.4, 153.6, 153.1, 151.8, 143.5, 139.6, 139.1, 136.6, 129.4, 128.9, 128.8, 128.0, 119.6, 117.0, 72.2, 68.2, 65.9, 38.7, 27.0, 23.0. HRMS $(M+H)^+$ calculated for $C_{27}H_{29}O_5N_8^+$ $(M+H)^+$ 545.2255, found 545.2257.

(*E*)-(6-(Benzyloxy)-2-(((cyclooct-2-en-1-yloxy)carbonyl)amino)-9*H*-purin-9-yl)methyl pivalate (6). Compound **2** (25 mg, 0.07 mmol) was dissolved in anhydrous DCM (1.4 mL) and anhydrous pyridine (0.06 mL, 4.3% v/v) under argon. This solution was cooled to 0 °C and phosgene (0.06 mL, 0.09 mmol, 15% in toluene) was added dropwise under stirring, after which the reaction was left at 0 °C and slowly warmed to room temperature overnight. Then a solution of (*E*)-cyclooct-2-en-1-ol (15 mg, 0.12 mmol) in anhydrous DCM (1.0 mL) was added to the reaction where it stirred at room temperature for an additional 3 hours. The volatiles were removed *in vacuo* and the resulting crude solid was purified using flash column chromatography on SiO_2 (25% EtOAc in Hex) to yield **6** (21 mg, 58%) as a colorless oil. ^{1}H NMR (500 MHz, $CDCl_3$) δ 8.02 (s, 1 H), 7.55 (d, 2 H, J = 7.1 Hz), 7.33 (m, 3 H), 6.09 (s, 2H), 5.98 (td, 1 H, J = 5.2, 3.7 Hz), 5.64 (s, 2 H), 5.58 (td, 2 H, J = 9.4, 2.2 Hz), 2.50 (m, 1 H), 2.20 (dd, 1 H, J = 6.4, 4.2 Hz), 2.4 (m, 4 H), 1.91 (m, 1 H), 1.74 (m, 2 H), 1.51 (m, 1 H), 1.16 (s, 9 H), 0.86 (m, 2 H). ^{13}C NMR (125 MHz, $CDCl_3$) δ 178.4, 171.3, 161.1, 160.1, 153.4, 152.7, 152.6, 151.2, 151.0, 142.1, 136.0, 132.5, 131.6, 130.7, 130.5, 130.1 128.8, 128.7, 128.6, 128.3, 127.8, 117.6, 75.1, 69.0, 68.9 64.2, 40.8, 38.9, 36.1, 36.0, 29.2, 26.9, 26.5, 24.3, 21.2. HRMS (ESI^+) calculated for $C_{27}H_{34}O_5N_5^-$ $(M-H)^+$ 508.2555, found 508.2546.

(6-(Benzyloxy)-2-(((1-(6-nitrobenzo[*d*][1,3]dioxol-5-yl)ethoxy)carbonyl)amino)-9H-purin-9-yl)methyl pivalate (7). Compound **2** (30 mg, 0.085 mmol) was dissolved in anhydrous DCM (2.0 mL) and anhydrous pyridine (85 μL) under argon. This solution was cooled to 0 °C and phosgene (0.122 mL, 0.17 mmol, 2.0 eq, 15% in toluene) was added dropwise under stirring, after which

the reaction was left at 0 °C, wrapped in foil, and slowly warmed to room temperature overnight. Then a solution of the caging group (21 mg, 0.10 mmol, 1.2 eq) in anhydrous DCM (0.6 mL) was added to the reaction where it stirred at room temperature for an additional 2 hours. The volatiles were removed *in vacuo* and the resulting crude solid was purified using flash column chromatography on SiO_2 (3% MeOH in DCM) to yield **7** (32 mg, 61%) as an off-white solid. ^{1}H NMR (500 MHz, d_6-DMSO) δ 10.59 (s, 1 H), 8.29 (s, 1 H) 7.65 (s, 1 H), 7.66 (s, 2 H), 7.56 (d, 2 H, *J* = 6.4 Hz), 7.42 (s, 1 H), 7.39 (m, 3 H), 6.24 (m, 3 H), 6.08 (s, 2 H), 5.60 (s, 2 H), 1.60 (d, 3 H, *J* = 6.4 Hz), 1.09 (s, 9 H). ^{13}C NMR (125 MHz, d_6-DMSO) δ 177.4, 160.3, 153.6, 152.9, 152.8, 151.4, 150.1, 147.6, 143.6, 141.5, 136.5, 135.5, 129.4, 128.9, 128.8, 124.4, 117.1, 106.4, 105.2, 104.1, 68.5, 68.4, 65.8, 38.7, 27.0, 22.4. HRMS $(M+H)^+$ calculated for $C_{28}H_{29}O_9N_6^+$ $(M+H)^+$ 593.1991, found 593.1994.

(6-(Benzyloxy)-2-(((1-(4-(4,4,5,5-tetramethyl-1,3,2-dioxaborolan-2-yl)phenyl)ethoxy)carbonyl)amino)-9H-purin-9-yl)methyl pivalate (8). Compound **2** (100 mg, 0.28 mmol) was dissolved in anhydrous DCM (6.4 mL) and anhydrous pyridine (0.280 mL) under argon. This solution was cooled to 0 °C and phosgene (0.400 mL, 0.56 mmol, 2.0 eq, 15% in toluene) was added dropwise under stirring, after which the reaction was left at 0 °C and slowly warmed to room temperature overnight. Then a solution of the caging group (84 mg, 0.34 mmol, 1.2 eq) in anhydrous DCM (2.0 mL) was added to the reaction where it stirred at room temperature for an additional 2 hours. The volatiles were removed *in vacuo* and the resulting crude solid was purified using flash column chromatography on SiO_2 (60% EtOAc in hexanes) to yield **8** (79 mg, 45%) as an off-white solid. ^{1}H NMR (500 MHz, d_6-DMSO) δ 10.52 (s, 1 H), 8.28 (s, 1 H), 7.70 (d, 2 H, *J* = 8.0 Hz), 7.56, (d, 2 H, *J* = 8.1 Hz), 7.49 (d, 2 H, *J* = 8.0 Hz), 7.38 (m, 3 H), 6.08 (s, 2 H), 5.87 (q, 1 H, *J* = 7.2 Hz), 5.60 (s, 2 H), 1.53 (d, 3 H, *J* = 6.7 Hz), 1.30 (s, 12 H), 1.09 (s, 9 H). ^{13}C NMR (125 MHz, d_6-DMSO) δ 177.4, 160.2, 153.6, 153.0, 151.8, 146.1, 143.5, 136.6, 135.0, 129.5, 128.9, 128.8, 125.6, 117.0, 84.1, 72.6, 68.3, 65.9, 38.7, 27.0, 25.4, 25.1, 23.1. HRMS (ESI^-) calculated for $C_{33}H_{39}O_7N_5B^-$ $(M-H)^-$ 628.2937, found 628.2940.

(2*R*,3*S*,4*R*,5*R*,6*R*)-2-(4-((((6-(Benzyloxy)-9-((pivaloyloxy)methyl)-9*H*-purin-2-yl)carbamoyl)oxy)methyl)-2-nitrophenoxy)-6-(methoxycarbonyl)tetrahydro-2*H*-pyran-3,4,5-triyl triacetate (9). Compound **2** (20 mg, 0.06 mmol) was dissolved in anhydrous DCM (1.4 mL) and anhydrous pyridine (0.060 mL) under argon. This solution was cooled to 0 °C and phosgene (0.085 mL, 0.18 mmol, 15% in toluene) was added dropwise under stirring, after which the reaction was left at 0 °C and slowly warmed to room temperature overnight. Then a solution of (2*S*,3*R*,4*S*,5*S*,6*S*)-2-(4-(hydroxymethyl)-2-nitrophenoxy)-6-(methoxycarbonyl)tetrahydro-2*H*-pyran-3,4,5-triyl triacetate (34 mg, 0.07 mmol) in anhydrous DCM (1.0 mL) was added to the reaction where it stirred at room temperature for 2 h. The volatiles were removed *in vacuo* and the resulting crude solid was purified using flash column chromatography on SiO_2 (2% MeOH in DCM) to yield **9** (47 mg, 91%) as a white solid. ^{1}H NMR (500 MHz, d_6-DMSO) δ 10.64 (s, 1 H), 8.28 (s, 1 H), 8.02 (d, *J* = 2.0 Hz, 1 H), 7.78 (dd, *J* = 9.4, 2.0 Hz, 1 H), 7.56 (d, *J* = 8.1 Hz, 2 H), 7.46 (d, *J* = 8.7 Hz, 1 H), 7.38 (m, 3 H), 6.07 (s, 2 H), 5.75 (d, *J* = 7.8 Hz, 1 H), 5.61 (s, 2 H), 5.46 (t, *J* = 9.4 Hz, 1 H), 5.22 (s, 2 H), 5.11 (m, 2 H), 4.74 (d, *J* = 9.8, 1 H), 3.64 (s, 3 H), 2.02 (m, 6 H), 2.00 (s, 3 H), 1.09 (s, 9 H). ^{13}C NMR (125 MHz, d_6-DMSO) 170.0, 169.3, 169.2, 166.7, 161.0, 153.2, 152.0, 151.0, 148.9, 142.1, 135.8, 133.6, 132.3, 128.7, 128.5, 128.4, 128.3, 124.9, 124.2, 120.3, 99.8, 72.6, 71.1, 70.2, 68.9, 68.7, 65.4, 64.2, 53.1, 38.8, 26.8, 20.6, 20.5, 20.5. HRMS $(M+H)^+$ calculated for $C_{39}H_{43}O_{17}N_6^+$ $(M+H)^+$ 867.2679, found 867.2701.

Allyl (6-(benzyloxy)-9H-purin-2-yl)carbamate (10). The protected compound **4** (50 mg, 0.11 mmol) was dissolved in EtOH (3.0 mL) and cooled to 0 °C. Ice cold 1 M NaOH (0.5 mL) was added dropwise and the reaction was stirred for 1 hour at 0 °C. Once complete by TLC, the reaction was neutralized with 10% acetic acid, the volatiles were evaporated *in vacuo*, and the resulting crude residue was purified through flash column chromatography on SiO_2 (5% MeOH in DCM) to yield **10** (17 mg, 48%) as a white solid. ^1H NMR (500 MHz, d_6-DMSO) δ 13.20 (s, 1 H), 10.29 (s, 1 H), 8.18 (s, 1 H), 7.59 (d, 2 H, *J* = 7.0 Hz), 7.38 (m, 3 H), 5.99 (ddt, 1 H, *J* = 17.2 10.5, 5.3 Hz), 5.61 (s, 2 H), 5.41 (dd, 1 H, *J* = 17.2, 1.7 Hz), 5.25 (dd, 1 H, *J* = 10.5, 1.7 Hz), 4.64 (d, 2 H, *J* = 5.3 Hz). ^{13}C NMR (125 MHz, d_6-DMSO) δ 152.3, 136.9, 133.7, 129.4, 128.9, 128.7, 117.9, 67.9, 65.1. HRMS $(M+H)^+$ calculated for $C_{16}H_{16}O_3N_5^+$ $(M+H)^+$ 326.1248, found 326.1256.

4-Azidobenzyl (6-(benzyloxy)-9H-purin-2-yl)carbamate (11). The protected compound **5** (60 mg, 0.11 mmol) was dissolved in EtOH (3.0 mL) and cooled to 0 °C. Ice cold 1 M NaOH (0.5 mL) was added dropwise and the reaction was stirred for 1 hour at 0 °C. Once complete by TLC, the reaction was neutralized with 10% acetic acid and the resulting precipitate was filtered, washed with cold ether, and dried to yield **11** (35 mg, 74%) as an off-white solid. ^1H NMR (500 MHz, d_6-DMSO) δ 13.18 (s, 1 H), 10.27 (s, 1 H), 8.24 (s, 1 H), 7.56 (d, 2 H, *J* = 6.8 Hz), 7.51 (d, 2 H, *J* = 8.5 Hz), 7.37 (m, 3 H), 7.14 (d, 2 H, *J* = 8.5 Hz), 5.84 (q, 1 H, *J* = 6.4 Hz), 5.57 (s, 2 H), 1.53 (d, 3 H, *J* = 6.4 Hz). ^{13}C NMR (125 MHz, d_6-DMSO) δ 151.9, 139.6, 139.1, 136.8, 129.4, 128.9, 128.7, 128.1, 119.6, 71.9, 67.9, 22.9. HRMS $(M+H)^+$ calculated for $C_{21}H_{19}O_3N_8^+$ $(M+H)^+$ 431.1575, found 431.1554.

(*E*)-Cyclooct-2-en-1-yl (6-(benzyloxy)-9*H*-purin-2-yl)carbamate (12). A solution of **6** (24 mg, 0.05 mmol) dissolved in EtOH (1.8 mL) was cooled to 0 °C. Ice cold 1 M NaOH (0.23 mL, 17% v/v) was added dropwise and the reaction was stirred for 1 hour at 0 °C. Once starting material was completely consumed as shown by thin layer chromatography, the reaction was neutralized with 10% acetic acid. However, no precipitate formed upon neutralization, so the volatiles were evaporated *in vacuo* and the crude residue was purified through flash column chromatography on SiO_2 (gradient, 60-80% EtOAc/Hex to yield the desired compound **12** (11 mg, 60%) as a colorless oil. ^1H NMR (500 MHz, $CDCl_3$) δ 8.25 (s, 1 H), 7.52 (d, 2 H, *J* = 7.2 Hz), 7.36 (dt, 3 H, *J* = 8.4, 7.3 Hz), 5.94 (td, 1 H, *J* = 5.2, 3.6 Hz), 5.63 (s, 2 H), 5.50 (s, 1 H), 2.50 (d, 1 H, *J* = 5.6 Hz), 2.18 (dd, 1 H, *J* = 6.3, 4.1 Hz), 2.06 (m, 2 H), 1.91 (m, 1 H), 1.81 (m, 1 H), 1.72 (m, 1 H), 1.52 (m, 1 H), 1.21 (m, 1 H), 1.15 (m, 1 H), 0.86 (m, 2 H). ^{13}C NMR (125 MHz, $CDCl_3$) δ 160.7, 160.7, 153.8, 151.3, 151.2, 143.1, 136.2, 132.6, 130.8, 130.6, 130.4, 128.62, 128.61, 128.3, 128.2, 128.15, 118.4, 75.3, 68.7, 40.7, 36.1, 36.1, 29.2, 27.3, 24.4, 23.5. HRMS (ESI^+) calculated for $C_{21}H_{22}O_3N_5^-$ $(M-H)^-$ 392.1724, found 392.1717.

1-(6-Nitrobenzo[*d*][1,3]dioxol-5-yl)ethyl (6-(benzyloxy)-9H-purin-2-yl)carbamate (13). The protected compound **7** (30 mg, 0.05 mmol) was dissolved in EtOH (1.4 mL) and cooled to 0 °C. Ice cold 1 M NaOH (0.230 mL) was added dropwise and the reaction was stirred for 1 hour at 0 °C. Once complete by TLC, the reaction was neutralized with 10% acetic acid and the resulting precipitate was filtered, washed with cold ether, and dried to yield **13** (17 mg, 71%) as an off-white solid. ^1H NMR (500 MHz, d_6-DMSO) δ 13.16 (s, 1 H), 10.36 (s, 1 H), 8.15 (s, 1 H), 7.63 (s, 1 H), 7.55 (d, 2 H, *J* = 7.2 Hz), 7.37 (m, 4 H), 6.22 (m, 3 H), 5.58 (s, 2 H), 1.59 (d, 3 H, *J* = 6.4 Hz). ^{13}C NMR (125 MHz, $CDCl_3$) δ 152.9, 147.6, 141.6, 134.6, 129.3, 129.4, 129.3, 128.9, 128.7, 106.3,

105.2, 104.0, 68.3, 68.0, 22.3. HRMS (ESI$^+$) calculated for $C_{22}H_{19}O_7N_6$ (M+H)$^+$ 479.1310, found 479.1306.

1-(4-(4,4,5,5-tetramethyl-1,3,2-dioxaborolan-2-yl)phenyl)ethyl (6-(benzyloxy)-9H-purin-2-yl)carbamate (14). The protected compound **8** (80 mg, 0.13 mmol) was dissolved in EtOH (3.5 mL) and cooled to 0 °C. Ice cold 1 M NaOH (0.590 mL) was added dropwise and the reaction was stirred for 1 hour at 0 °C. Once complete by TLC, the reaction was neutralized with 10% acetic acid and the resulting precipitate was filtered, washed with cold ether, and dried to yield **14** (40 mg, 60%) as an off-white solid. ^{1}H NMR (500 MHz, d_6-DMSO) δ 10.28 (s, 1 H), 8.17 (s, 1 H), 7.69 (d, 2 H, *J* = 8.0 Hz), 7.55 (d, 2 H, *J* = 6.7 Hz), 7.46 (d, 2 H, *J* = 8.0 Hz), 7.37 (m, 3 H), 5.85 (q, 1 H, *J* = 6.6 Hz), 5.58 (s, 2 H), 1.53 (d, 3 H, *J* = 6.4 Hz), 1.29 (s, 12 H). ^{13}C NMR (125 MHz, d_6-DMSO) δ 152.0, 151.9, 146.1, 136.8, 135.0, 129.3, 128.9, 128.7, 125.6, 84.1, 72.2, 67.9, 25.1, 23.0. HRMS (M+H)$^+$ calculated for $C_{27}H_{31}BN_5O_5^+$ (M+H)$^+$ 516.2413, found 516.2415.

(4-(1-(((6-(Benzyloxy)-9H-purin-2-yl)carbamoyl)oxy)ethyl)phenyl)boronic acid (15). Compound **14** (20 mg, 0.039 mmol) and sodium periodate (25 mg, 0.12 mmol, 3.0 eq) were dissolved in a 4:1 mixture of THF and water (1.0 mL total) and stirred at room temperature for 30 minutes under argon. Then 1 M HCl (27 µL) was added to the suspension where it stirred for 18 hours at room temperature. Upon completion by TLC, the reaction mixture was diluted with water (3 mL) and extracted with DCM (3 x 5 mL). The combined extracts were washed with brine (3 mL), dried over Na_2SO_4, and concentrated *in vacuo*. The resulting crude residue was purified through flash column chromatography (5% MeOH in DCM) to yield the desired compound **15** (12 mg, 25%) as an off-white solid. ^{1}H NMR (500 MHz, d_6-DMSO) δ 10.28 (s, 1 H), 8.18 (s, 1 H), 7.79 (d, *J* = 8.0 Hz, 2 H), 7.56 (d, *J* = 7.0 Hz, 2 H), 7.39 (m, 5 H), 5.84 (q, *J* = 6.6 Hz, 1 H), 5.59 (s, 2 H), 1.54 (d, *J* = 6.6 Hz). ^{13}C NMR (125 MHz, d_6-DMSO) δ 152.3, 151.2, 136.8, 134.6, 134.5, 129.4, 128.9, 128.6, 125.3, 67.9, 30.9, 23.0. HRMS (ESI$^+$) calculated for $C_{21}H_{21}O_5N_5B^+$ (M+H)$^+$ 434.1630, found 434.1624.

(3*S*,4*S*,5*R*,6*S*)-6-(4-((((6-(Benzyloxy)-9H-purin-2-yl)carbamoyl)oxy)methyl)-2-nitrophenoxy)-3,4,5-trihydroxytetrahydro-2H-pyran-2-carboxylic acid (16). A solution of **9** (45 mg, 0.05 mmol) dissolved in EtOH (0.24 mL) was cooled to 0 °C. Ice cold 1 M NaOH (1.4 mL) was added dropwise, and the reaction was stirred for 1 hour at 0 °C. Once complete by TLC, the reaction was neutralized with 10% AcOH. Once complete by TLC, the reaction was neutralized with 10% acetic acid and the solution was dried, dissolved in 1:1 DMSO/H_2O and reverse-phase purified using a Agilent Prep-C18 (5 µM, 21.2 x 150 mm, P/N 443905-102), with a 20 minute gradient of 5-45% acetonitrile (0.1% TFA) and water (0.1% TFA). After lyophilization, **16** was isolated (15 mg, 47%) as slightly yellow solid. ^{1}H NMR (500 MHz, d_6-DMSO) δ 10.44 (s, 1 H), 8.28 (s, 1 H), 8.05 (s, 1 H), 7.8 (d, *J* = 8.8 Hz, 1 H), 7.63 (d, *J* = 6.8 Hz, 2 H), 7.54 (d, *J* = 8.8 Hz), 7.44 (m, 4 H), 5.66 (s, 2 H), 5.35 (d, *J* = 7.0 Hz, 1 H), 5.25 (s, 2 H), 4.00 (d, *J* = 10.3 Hz, 1 H), 3.7 (s, 1 H), 3.36 (m, 1 H). ^{13}C NMR (125 MHz, d_6-DMSO) δ 170.3, 159.3, 152.3, 152.2, 149.2, 142.1, 140.4, 136.7, 134.0, 131.3, 129.6, 128.9, 128.7, 124.6, 117.5, 115.1, 100.5, 76.3, 75.9, 73.2, 71.6, 68.1, 64.6, 52.2 HRMS (M-H)$^-$ calculated for $C_{26}H_{23}O_{12}N_6^-$ (M-H)$^-$ 611.1369, found 611.1365.

Biological Protocols

SNAP-tag Expression and Purification. pET51b-His-TEV-SNAP-tag_fast (Addgene, 167271) was transformed into BL21 chemically competent cells, and cells were plated on 10 mL of LB agar supplemented with 10 µL of a 10 mg/mL stock of ampicillin. A single colony was inoculated into 5 mL of LB broth supplemented with 5 µL of a 10 mg/mL ampicillin stock and grown overnight with shaking (37 ºC, 250 rpm). A 200 mL culture supplemented with 200 µL of a 10 mg/mL ampicillin stock was prepared by inoculating it with 2 mL of the saturated overnight culture and incubated with shaking (37 ºC, 250 rpm) until OD600 reached 0.8. Expression was induced by addition of 50 µL of 1 M IPTG in water, and the culture was incubated overnight at 17 ºC with shaking (250 rpm). Cells were harvested by transferring the culture to 4 x 50 mL conical tubes (Fisher Scientific, 05-539-8), followed by centrifugation at 3800 g for 10 minutes at 4 ºC, and the supernatant was discarded. Each pellet was resuspended in 4 mL of ice-cold lysis buffer (200 mM NaCl, 50 mM Tris-HCl, 10% glycerol, 5 mM ß-mercaptoethanol (BME), 1 mg/mL lysozyme, and 1 mM phenylmethylsulfonyl fluoride (PMSF) in milliQ water) and cooled on ice. Cell lysis was performed using a Fisher Scientific Model 550 Sonic Dismembrator. The suspension solution was sonicated in 20 second pulses at 20 second intervals for two minutes using power level three. Cellular debris was pelleted by centrifugation at 3800 g for 20 minutes and the supernatant was transferred to a fresh 15 mL conical tube (Fisher Scientific, 05-539-12). The lysate was incubated with 500 µL of Ni-NTA resin (G-Biosciences, 786939) for 2 hours at 4 ºC with rocking. The resin was gently pelleted by centrifugation at 1000 g for 10 minutes at 4 ºC. The supernatant was discarded, and the resin was washed three times with 500 µL of ice-cold wash buffer (200 mM NaCl, 50 mM Tris-HCl, 10% glycerol, 25 mM imidazole, 5 mM BME, milliQ water) with a 10 minute spin at 1000 g between each wash in which the supernatant was discarded. The protein was eluted from the resin with three consecutive 5 minute incubations with 200 µL of ice-cold elution buffer (200 mM NaCl, 50 mM Tris-HCl, 10% glycerol, 400 mM imidazole, 5 mM BME, milliQ water) with a 10 minute centrifugation step at 1000 g between each elution. The elution fractions were combined, and the purified protein was buffer exchanged using a 3 kDa centrifugal filter (Fisher Scientific, UFC500396) into storage buffer (50 mM Tris-HCl, pH 8, 200 mM NaCl, 30% glycerol). Protein concentration was determined using a Bradford assay (Thermo Fisher). The protein was diluted to 300 µM and stored in aliquots at –80 ºC until further use.

Production of primary human SNAP-CAR T cells. Primary human SNAP-CAR T cells were produced as in Supporting Reference 2. Buffy coat samples purchased from the Pittsburgh Central Blood Bank and leukopaks from Miltenyi Biotec fulfill the basic exempt criteria 45 CFR 46.101(b)(4) under the University of Pittsburgh IRB guidelines. Briefly, PBMC were isolated from Buffy Coats or leukopaks from healthy volunteer donors using Ficoll gradient centrifugation. Human T cells were isolated using the Human Pan T cell isolation kit (Cat# 130-096-535, Miltenyi Biotec) following guidelines from the manufacturer. Human T cells were cultured in supplemented RPMI media with 10% Human AB serum (Gemini Bio-Products), 100 U/mL human IL-2 IS (Miltenyi Biotec), 1 ng/mL IL-15 (Miltenyi Biotec), and 4mM L-Arginine (Sigma Aldrich). T cells were stimulated and expanded using TransAct Human T cell activation reagent (Cat# 130-128-758, Miltenyi Biotec). For transduction, 48 hours after activation, gamma retrovirus encoding SNAP-CAR was added to the isolated CD3-positive cells. Transduction was performed by spinning cells on retronectin (RN) (Takara Bio)-coated plates. To coat the plates, 10 µg/mL RN in PBS was added to a plate at 4 °C for 24 h. RN was removed and 2ml of viral supernatant was

added to each well and spun at 2000 x g for 2 h at 32 °C. After removing 2 mL of media, 1×10^6 cells in 4 mL were added per well and spun at 32 °C for 10 minutes at 1000 x g. The cells were cultured in an incubator maintained at 37 °C. Twenty-four hours post spinfection, 100 U/mL IL-2 and 1 ng/mL IL-15 were added to each well. Three days post the addition of cytokines, cells were split to a well in a new 6 well plate and maintained at a concentration of 1×10^6/mL, supplemented with fresh IL-2 and IL-15 every 2–3 days. Flow cytometry was performed on transduced cells by staining for LNGFR marker on gamma retrovirus using anti-LNGFR antibody (cat# 562663, BD Biosciences).

NMR Spectra

4, 500 MHz,
d$_6$-DMSO

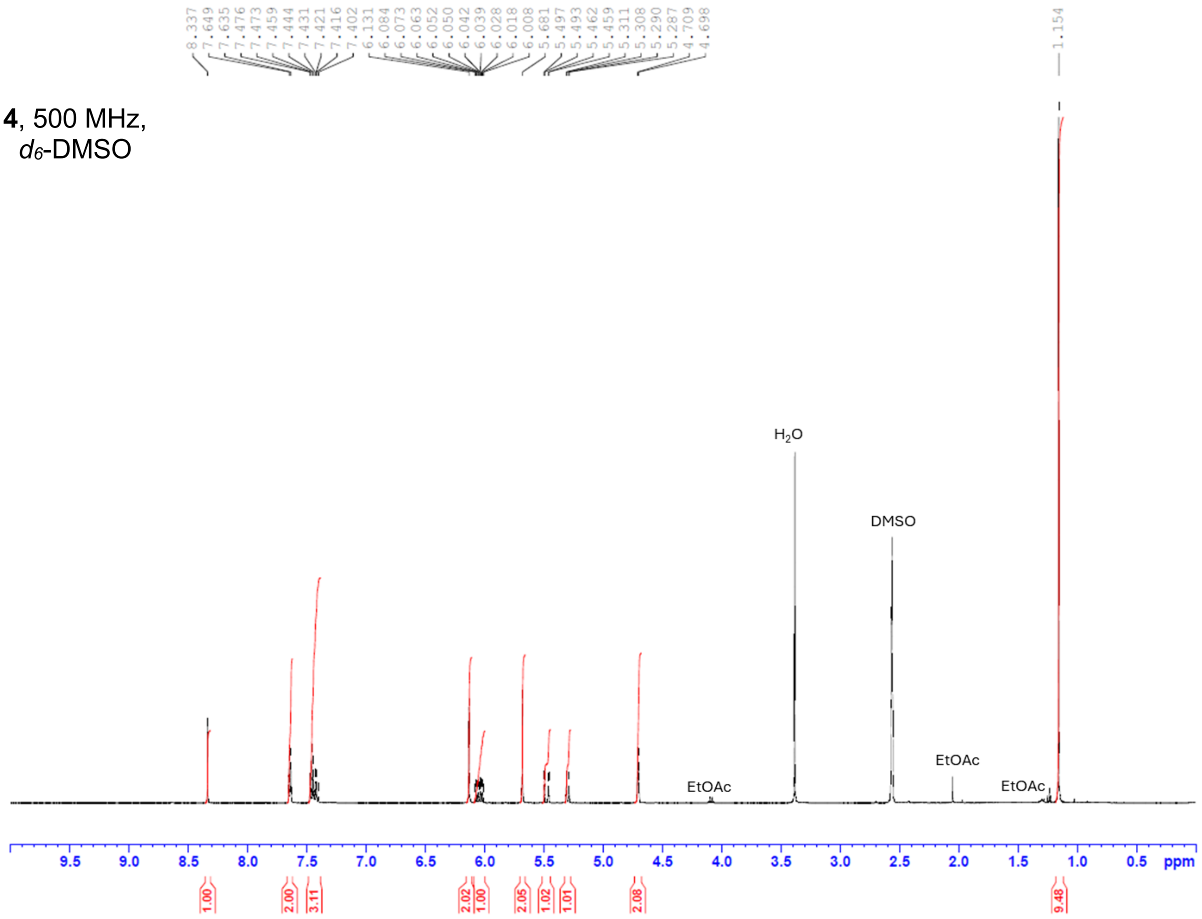

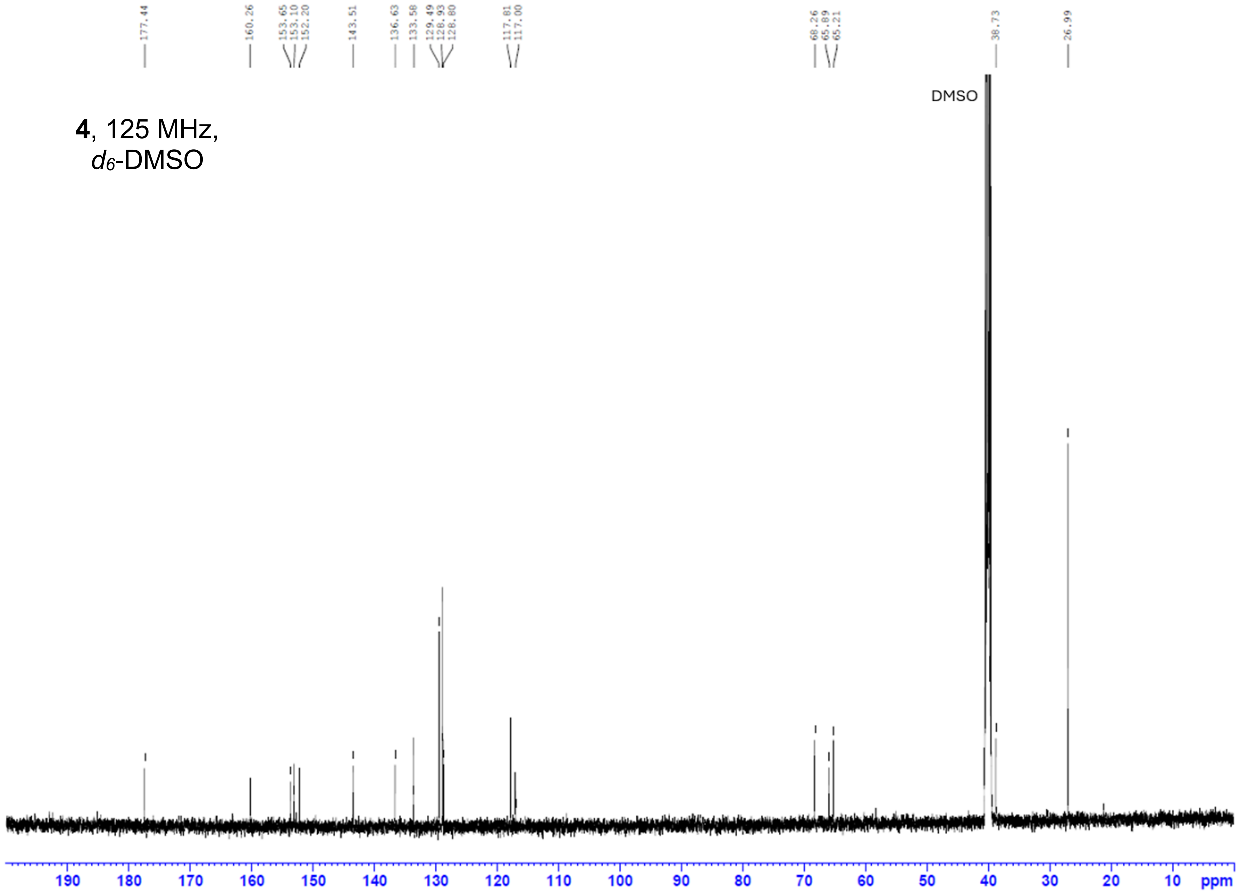
4, 125 MHz,
*d*6-DMSO
177.44
160.26
153.65
153.10
152.20
143.51
136.63
133.58
129.49
128.93
128.80
117.81
117.00
68.26
65.89
65.21
38.73
26.99
DMSO
190
180
170
160
150
140
130
120
110
100
90
80
70
60
50
40
30
20
10
ppm

5, 500 MHz,
*d*6-DMSO

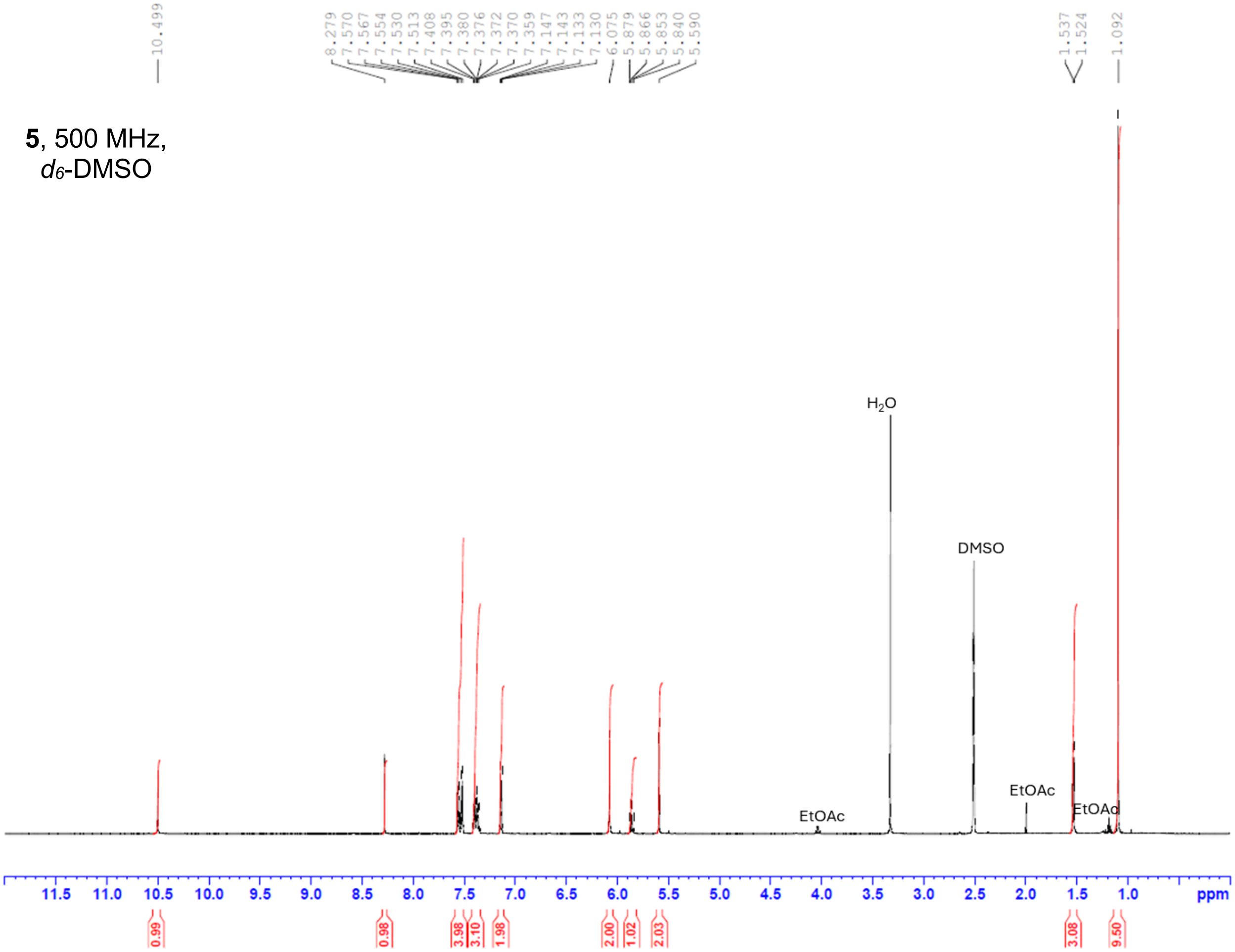

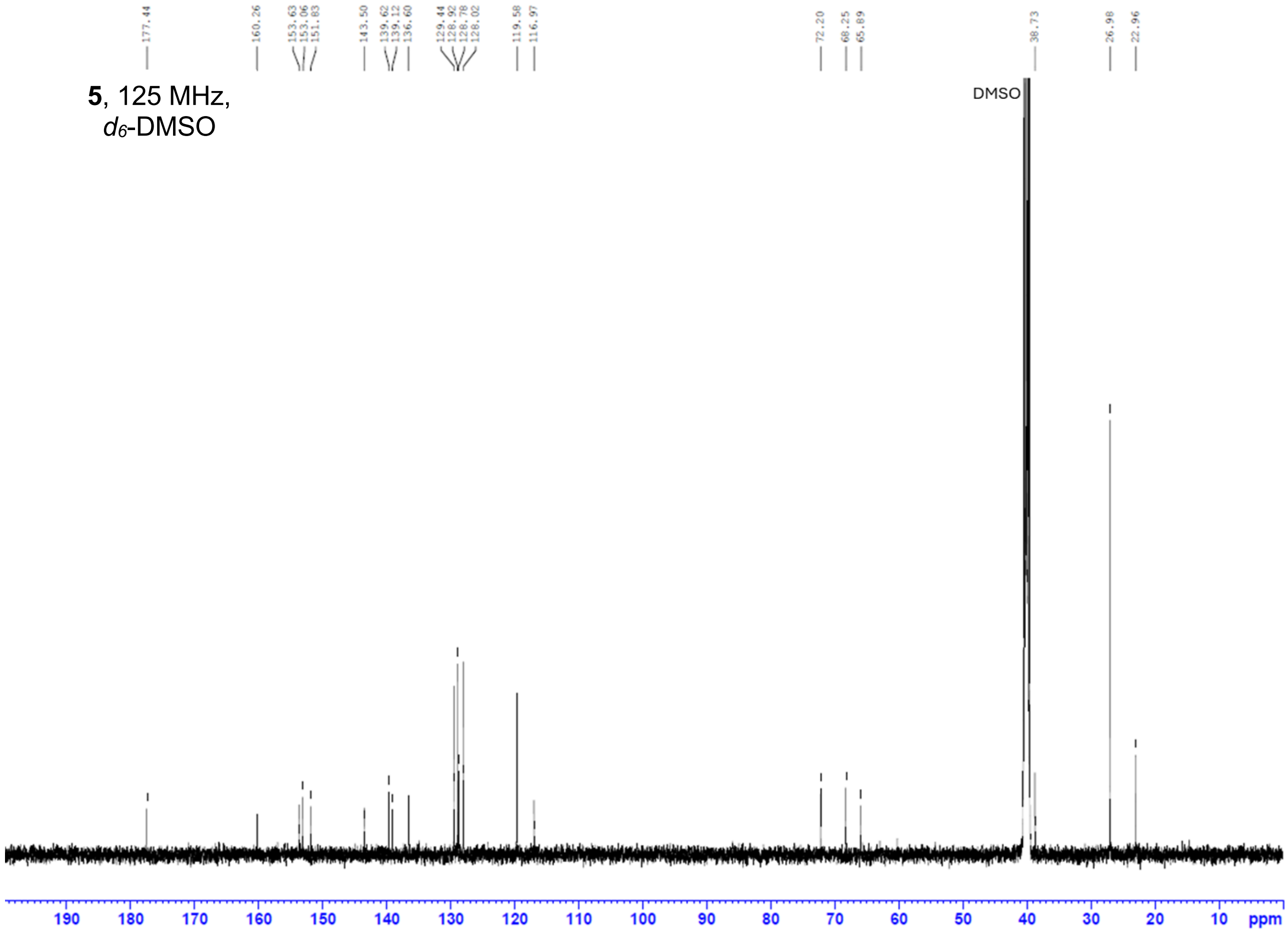

177.44
160.26
153.63
153.06
151.83
143.50
139.62
139.12
136.60
129.44
128.92
128.78
128.02
119.58
116.97
72.20
68.25
65.89
38.73
26.98
22.96
5, 125 MHz,
d6-DMSO
DMSO
190
180
170
160
150
140
130
120
110
100
90
80
70
60
50
40
30
20
10
ppm

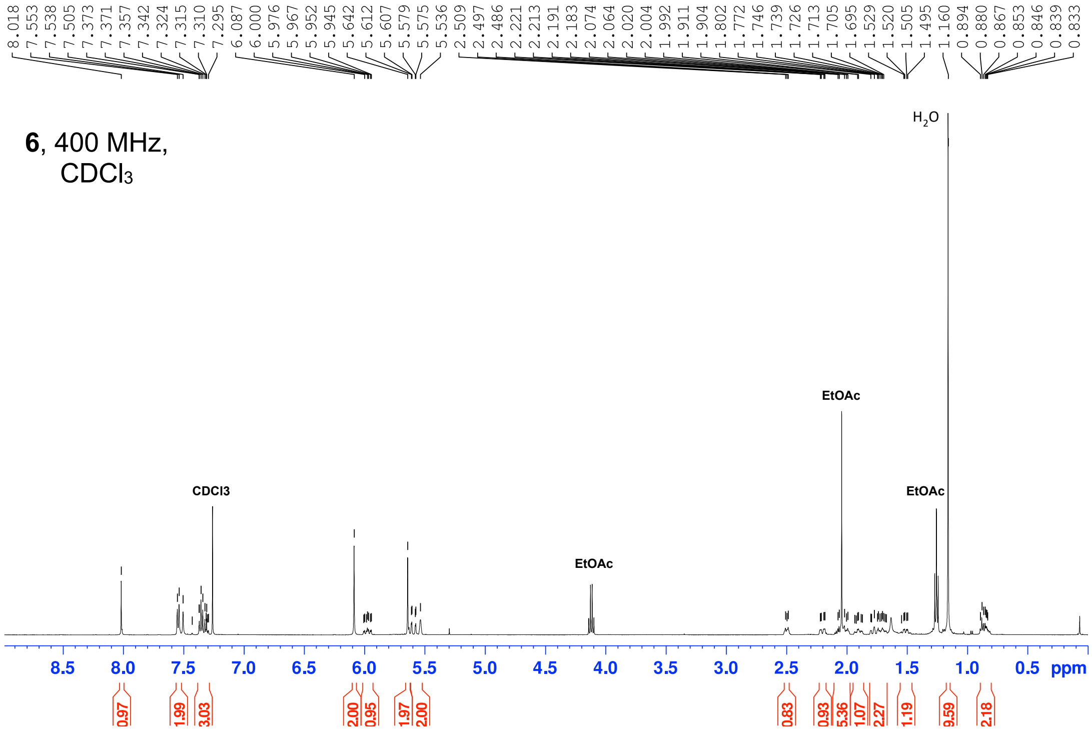
6, 400 MHz,
CDCl3
H2O
EtOAc
EtOAc
EtOAc
CDCl3
ppm

6, 100 MHz, $CDCl_3$

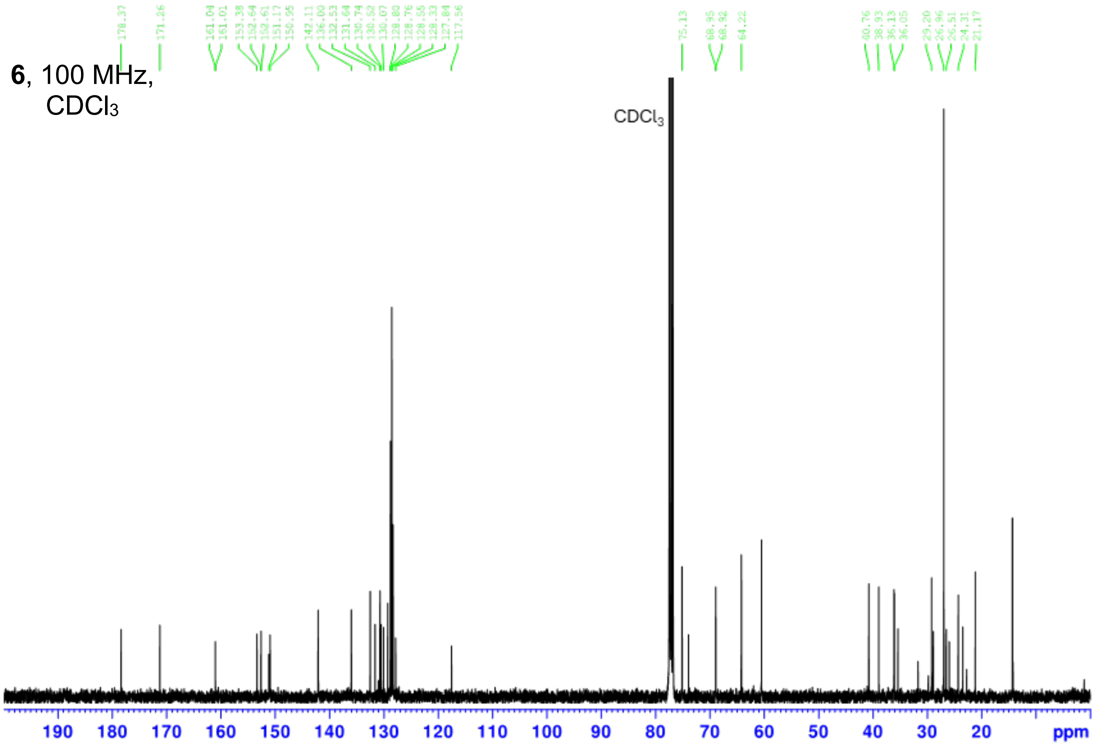

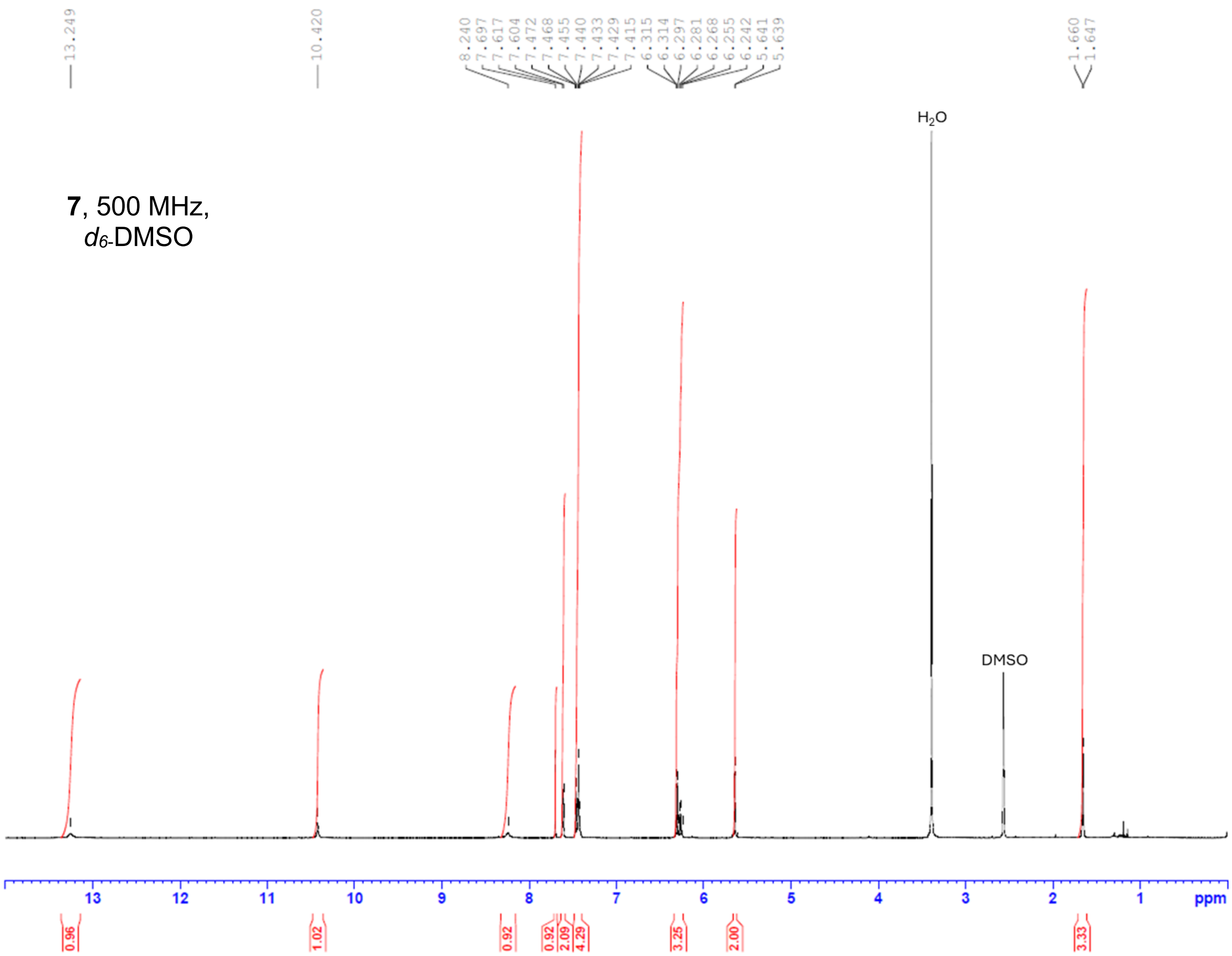

13.249
10.420
8.240
7.697
7.617
7.604
7.472
7.468
7.455
7.440
7.433
7.429
7.415
6.315
6.314
6.297
6.281
6.268
6.255
6.242
5.641
5.639
1.660
1.647
7, 500 MHz,
*d*6-DMSO
H2O
DMSO
13
12
11
10
9
8
7
6
5
4
3
2
1
ppm
0.96
1.02
0.92
0.92
2.09
4.29
3.25
2.00
3.33

7, 125 MHz, *d*6-DMSO

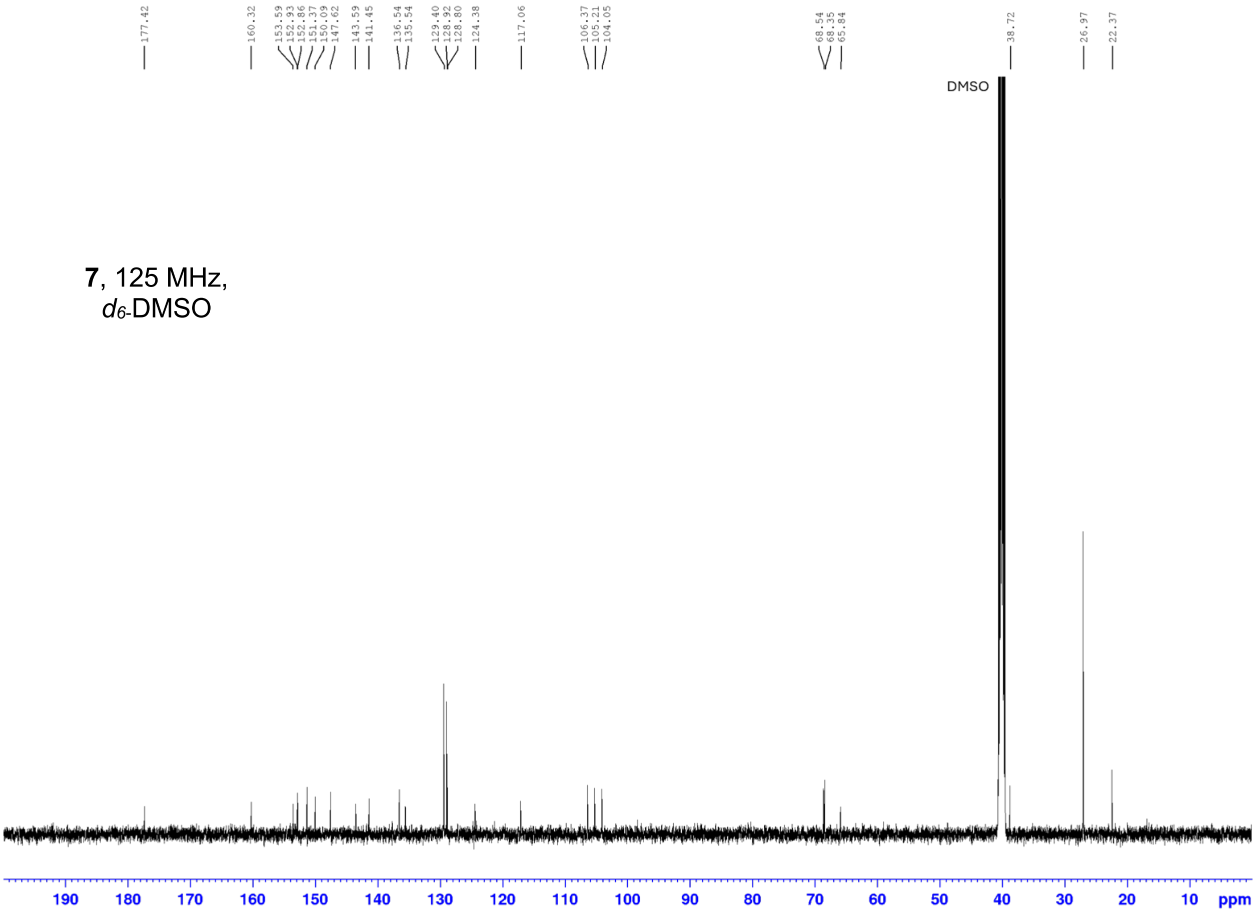

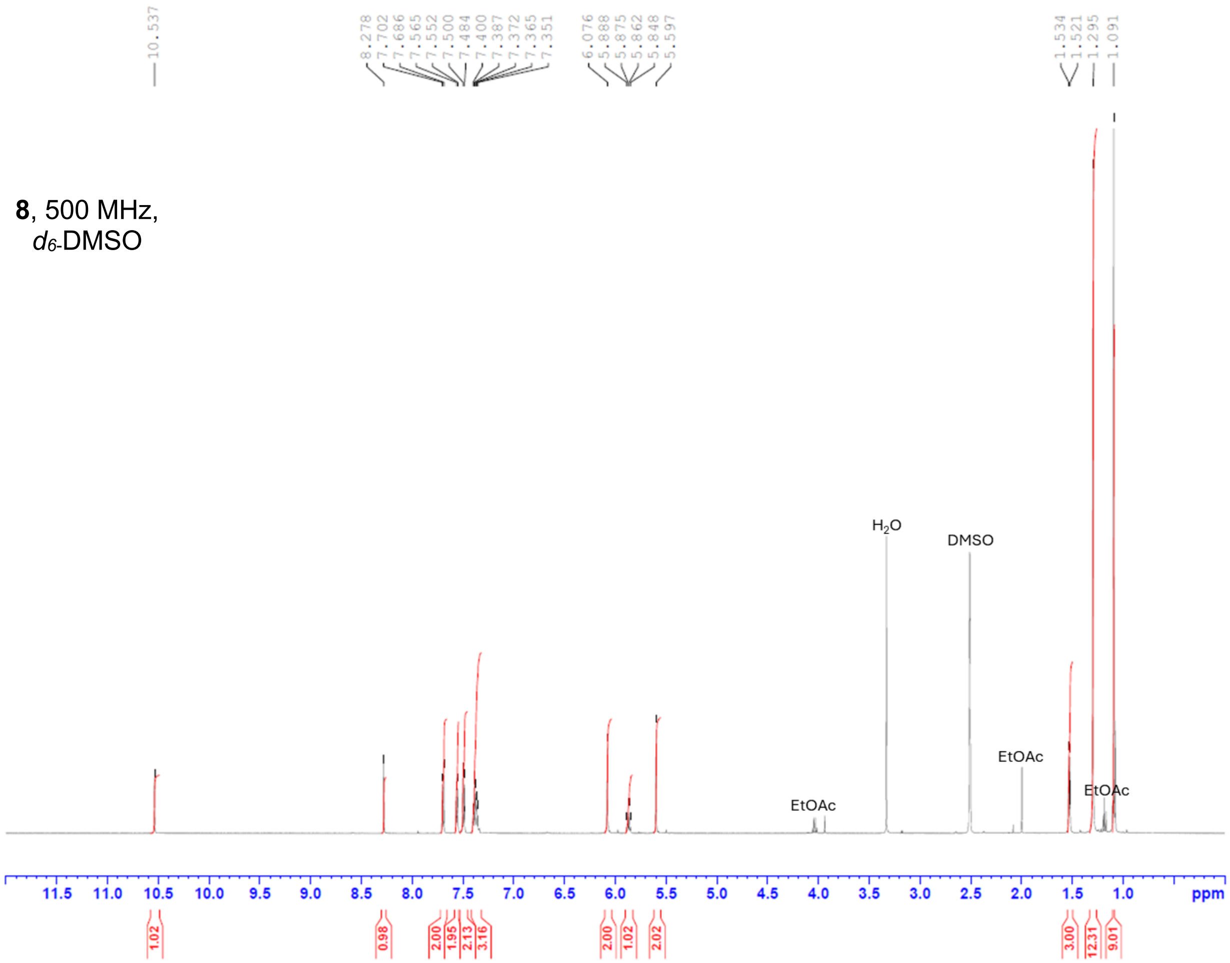
8, 500 MHz,
d_6-DMSO
10.537
8.278
7.702
7.686
7.565
7.552
7.500
7.484
7.400
7.387
7.372
7.365
7.351
6.076
5.888
5.875
5.862
5.848
5.597
1.534
1.521
1.295
1.091
H_2O
DMSO
EtOAc
EtOAc
EtOAc
11.5 11.0 10.5 10.0 9.5 9.0 8.5 8.0 7.5 7.0 6.5 6.0 5.5 5.0 4.5 4.0 3.5 3.0 2.5 2.0 1.5 1.0 ppm
1.02
0.98
2.00
1.95
2.13
3.16
2.00
1.02
2.02
3.00
12.31
9.01

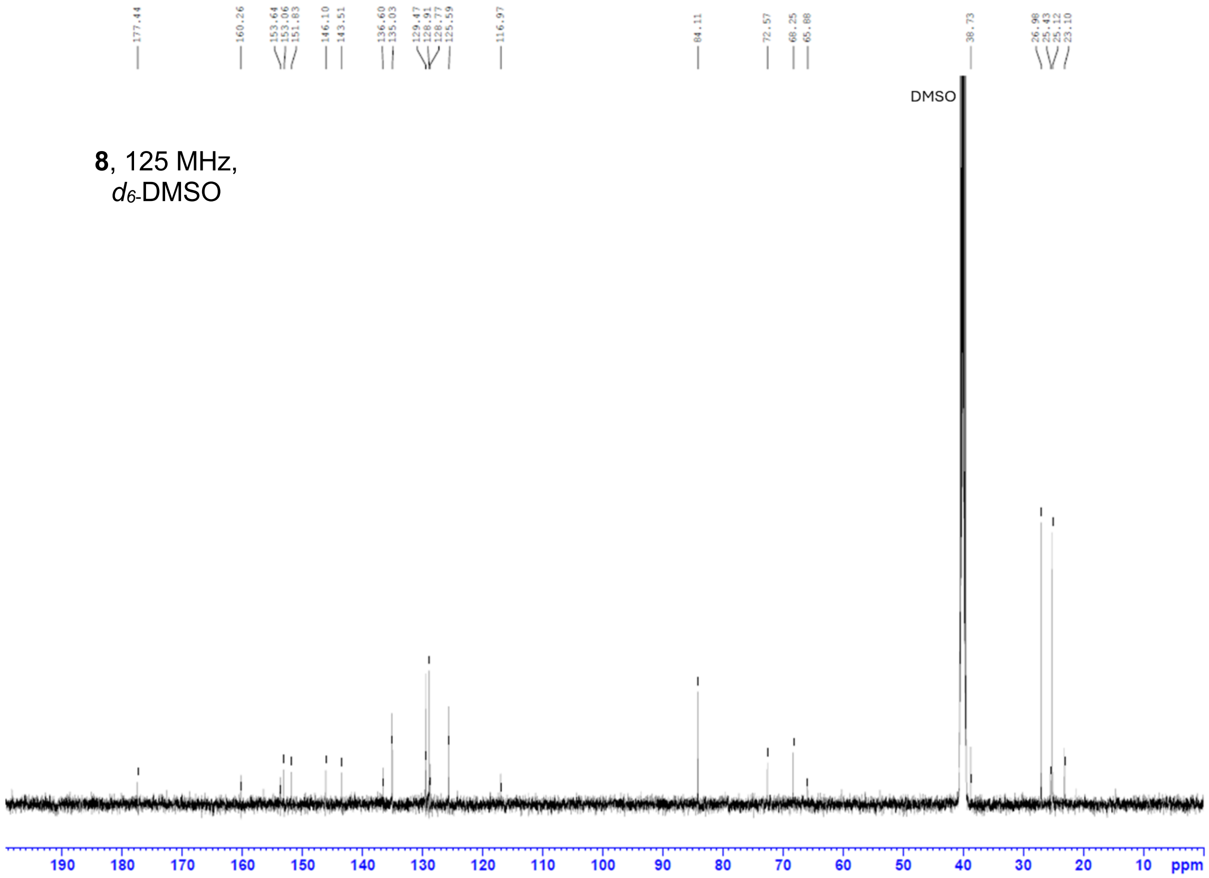

8, 125 MHz,
d_6-DMSO
DMSO
177.44
160.26
153.64
153.06
151.83
146.10
143.51
136.60
135.03
129.47
128.91
128.77
125.59
116.97
84.11
72.57
68.25
65.88
38.73
26.98
25.43
25.12
23.10
190 180 170 160 150 140 130 120 110 100 90 80 70 60 50 40 30 20 10 ppm

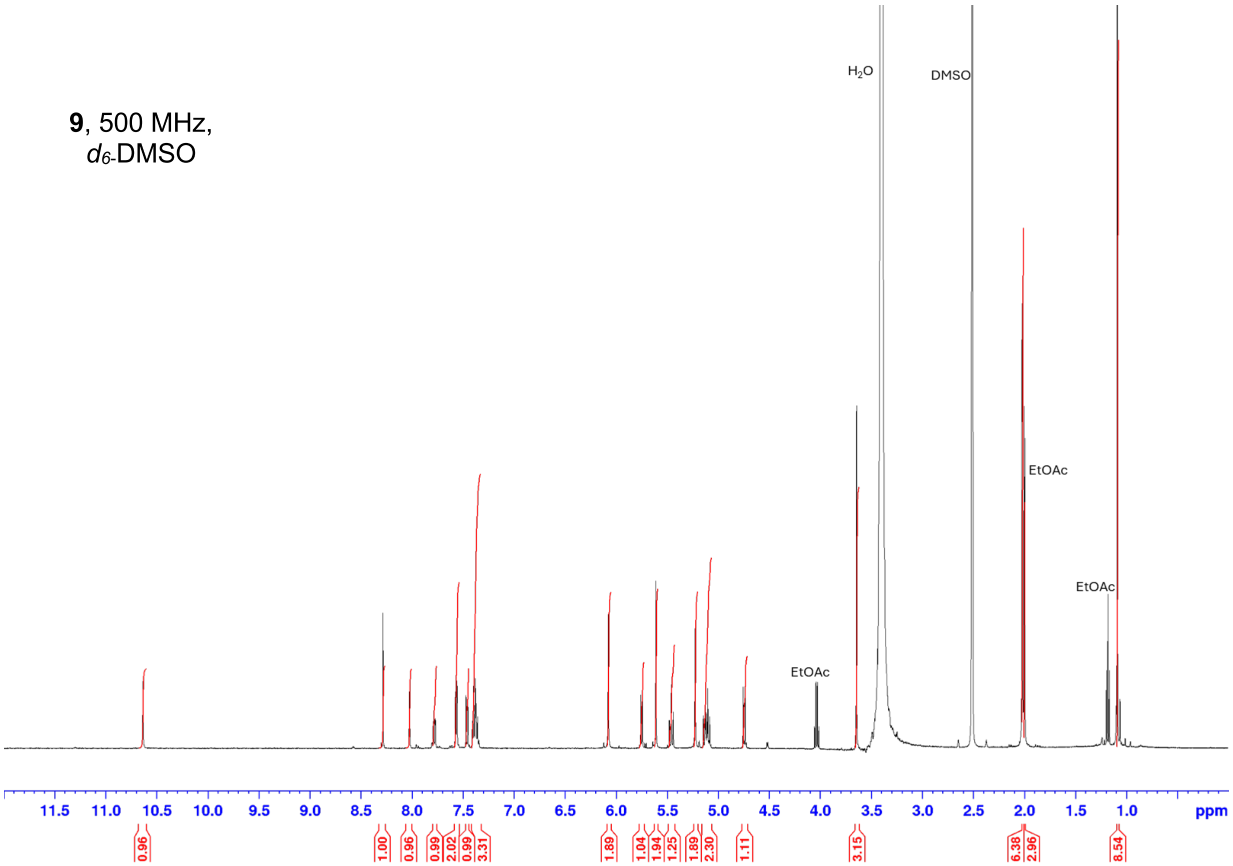

9, 500 MHz,
*d*6-DMSO
H2O
DMSO
EtOAc
EtOAc
EtOAc
11.5 11.0 10.5 10.0 9.5 9.0 8.5 8.0 7.5 7.0 6.5 6.0 5.5 5.0 4.5 4.0 3.5 3.0 2.5 2.0 1.5 1.0 ppm
0.96
1.00
0.96
0.99
2.02
0.99
3.31
1.89
1.04
1.94
1.25
1.89
2.30
1.11
3.15
6.38
2.96
8.54

9, 125 MHz, d_6-DMSO

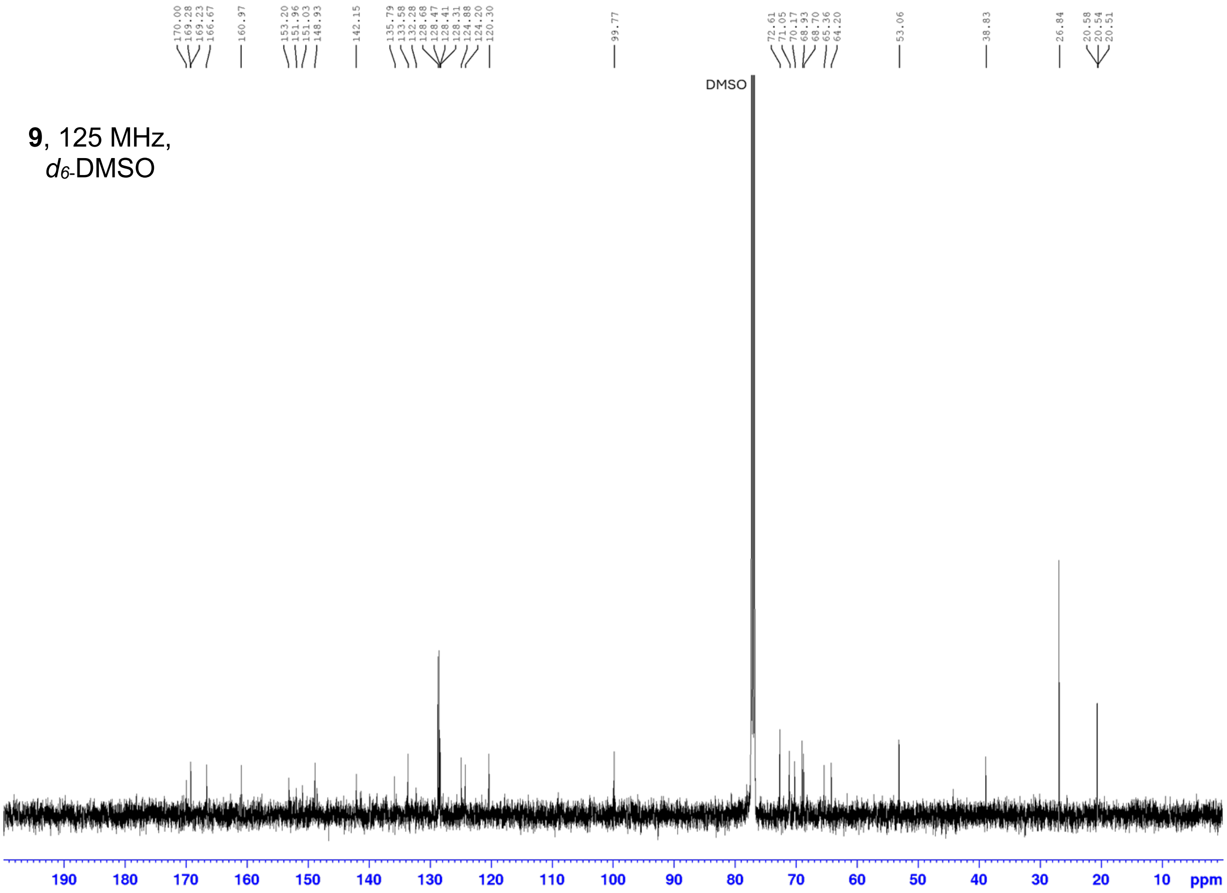

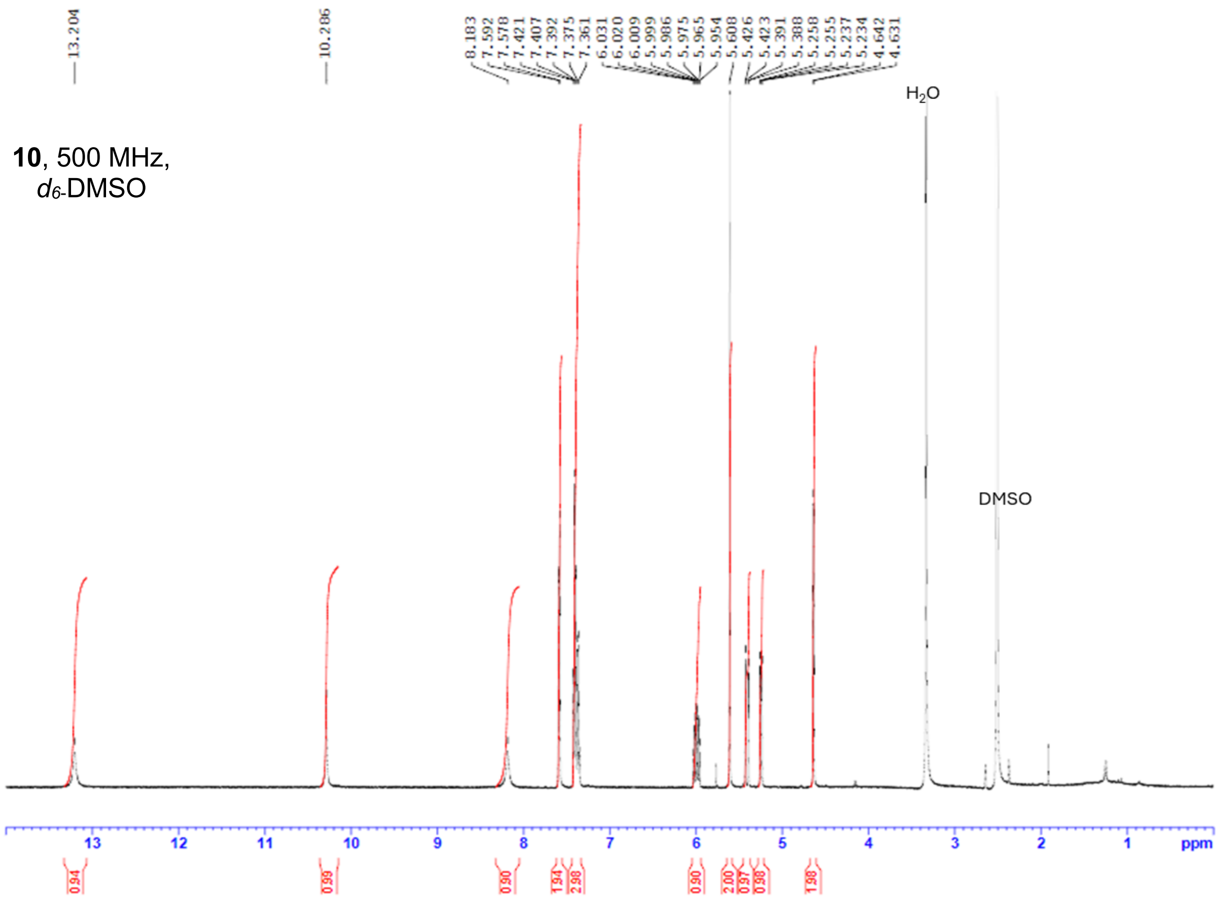
10, 500 MHz,
d6-DMSO
13.204
10.286
8.183
7.592
7.578
7.421
7.407
7.392
7.375
7.361
6.031
6.020
6.009
5.999
5.986
5.975
5.965
5.954
5.608
5.426
5.423
5.391
5.388
5.258
5.255
5.237
5.234
4.642
4.631
H2O
DMSO
13
12
11
10
9
8
7
6
5
4
3
2
1
ppm
0.94
0.99
0.90
1.94
2.98
0.90
2.00
0.97
0.98
1.98

10, 125 MHz,
d_6-DMSO

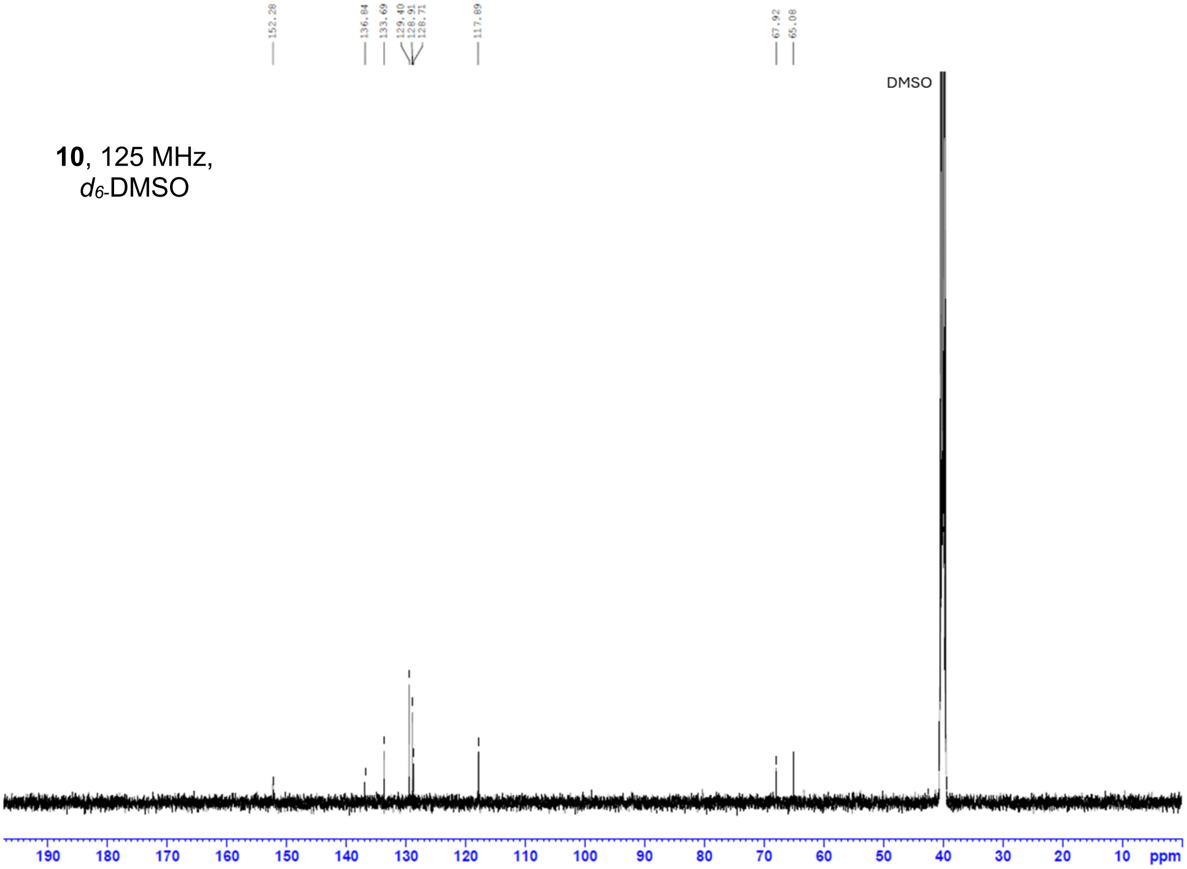

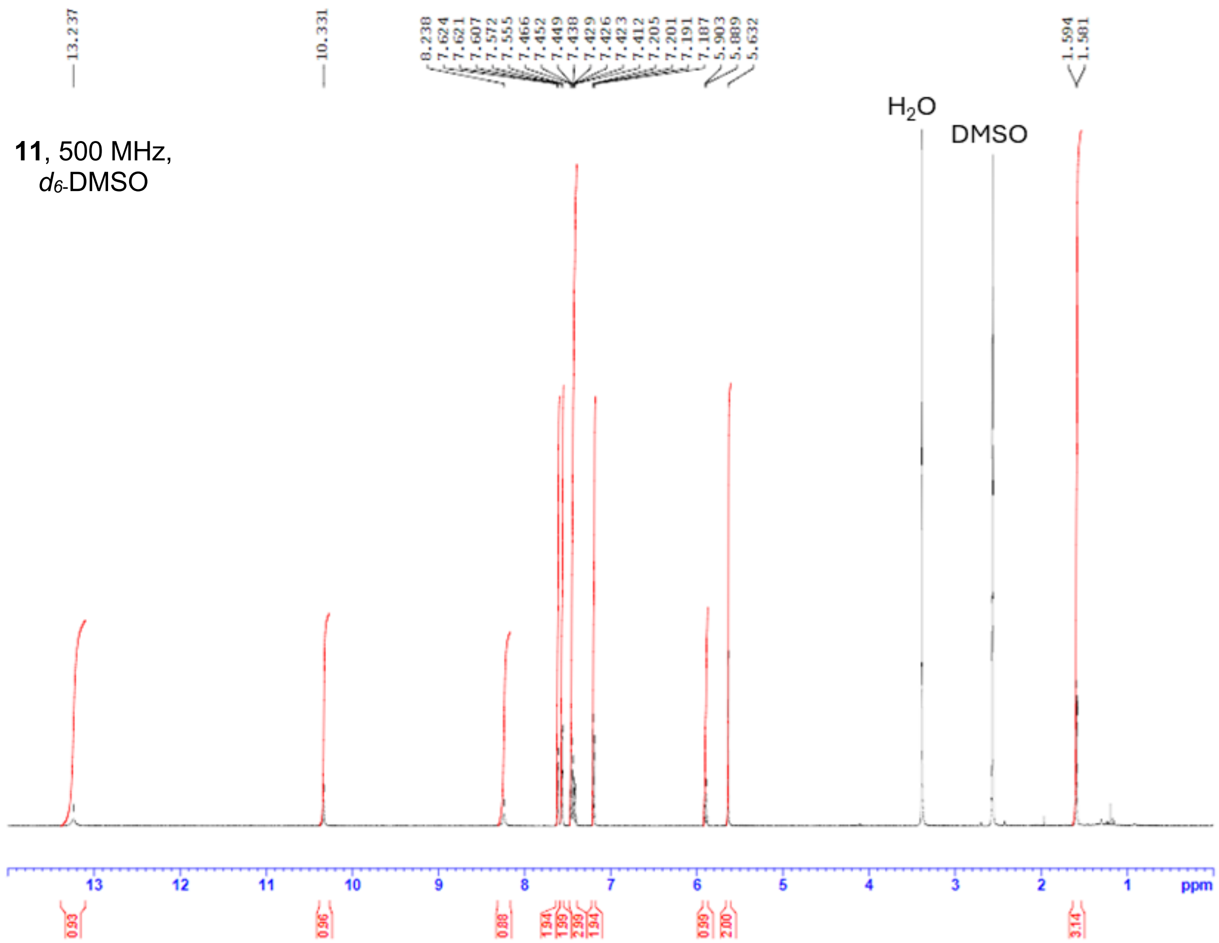

11, 500 MHz,
*d*6-DMSO
H2O
DMSO
13.237
10.331
8.238
7.624
7.621
7.607
7.572
7.555
7.466
7.452
7.449
7.438
7.429
7.426
7.423
7.412
7.205
7.201
7.191
7.187
5.903
5.889
5.632
1.594
1.581
ppm
0.93
0.96
0.88
1.94
1.99
2.99
1.94
0.99
2.00
3.14

11, 125 MHz, d_6-DMSO

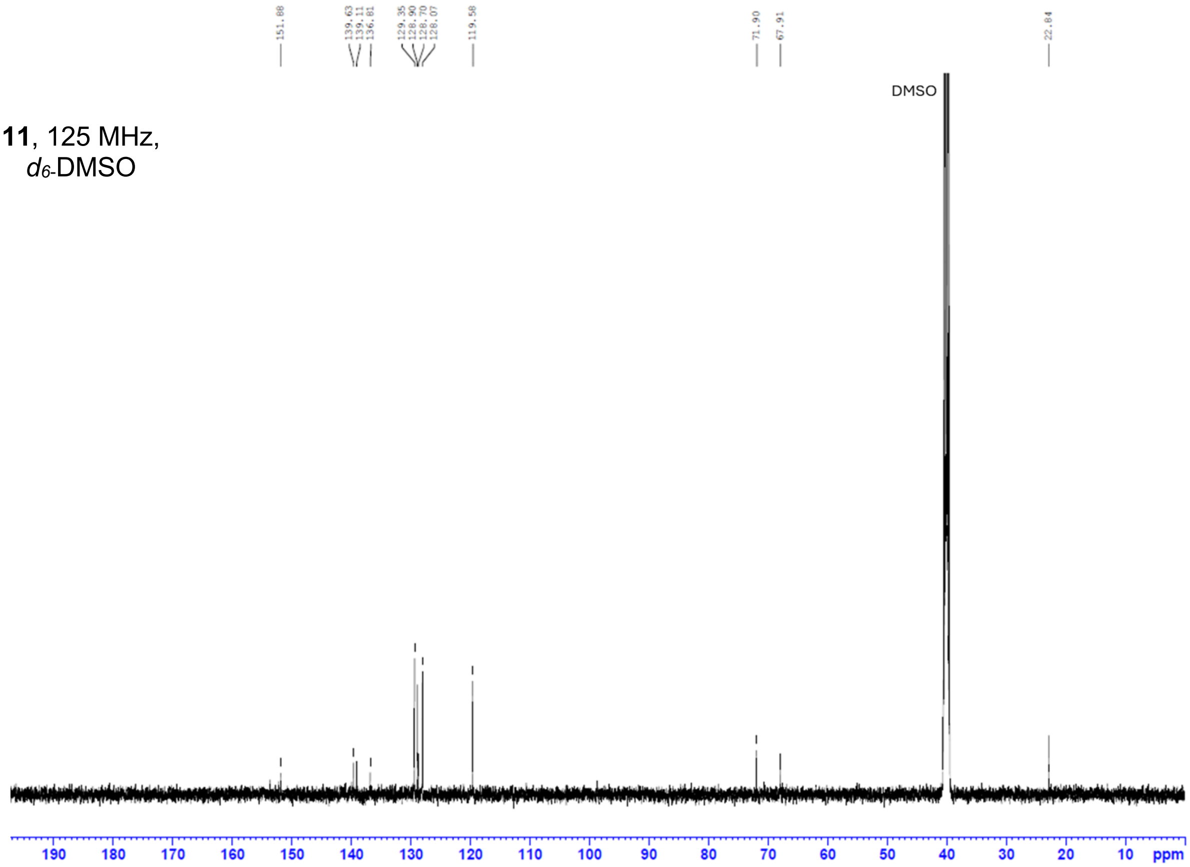

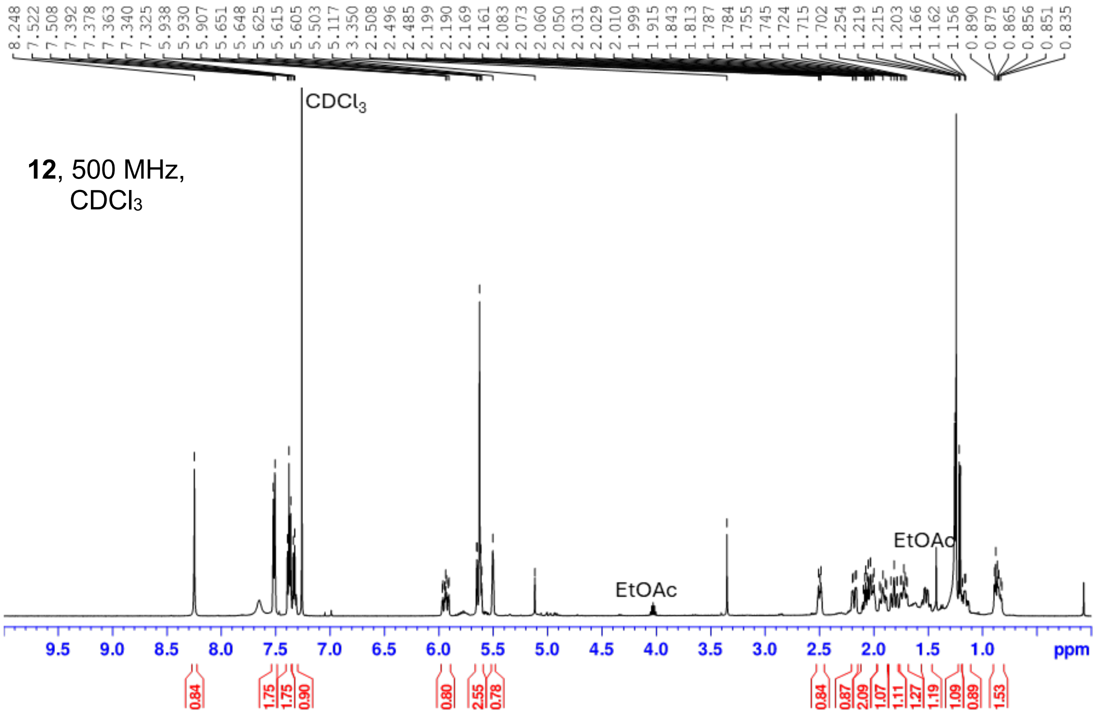

12, 5125 MHz,
CDCl$_3$

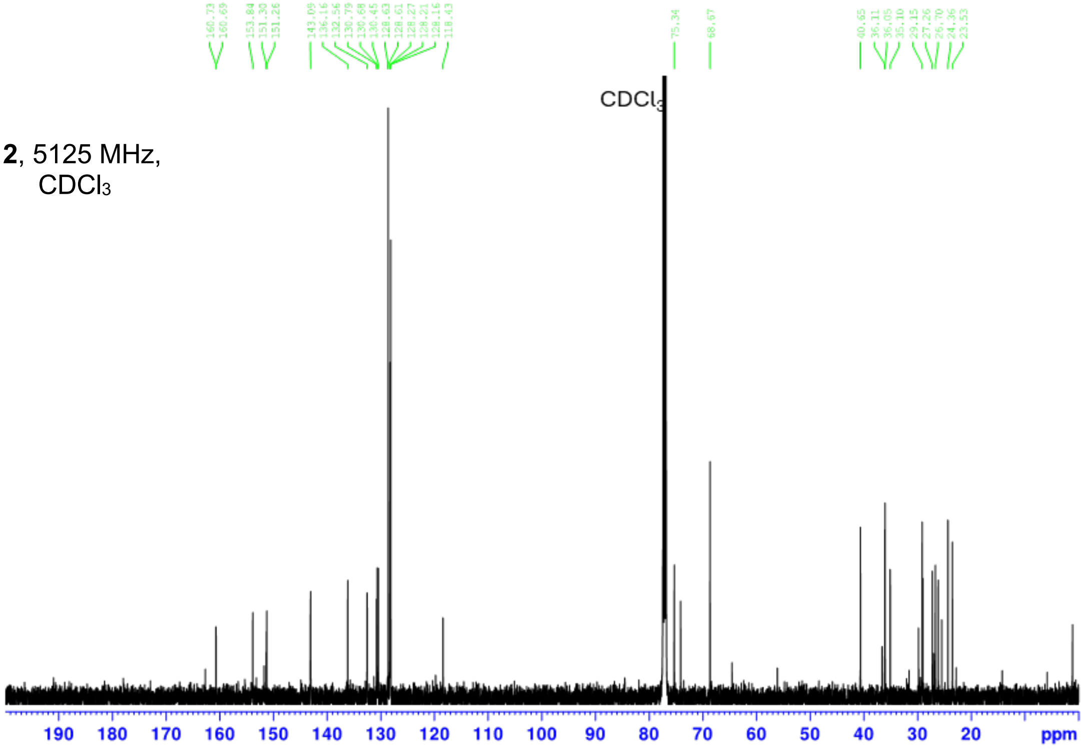

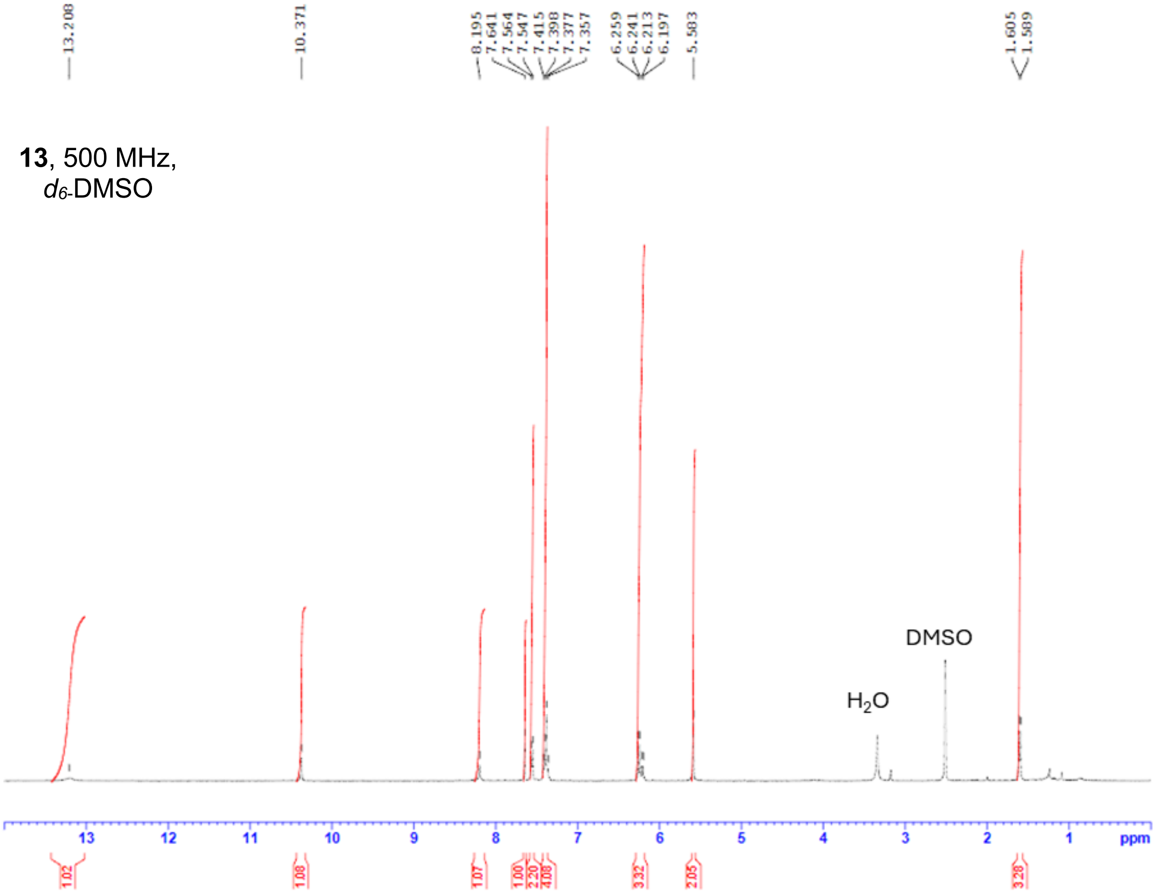
13.208
10.371
8.195
7.641
7.564
7.547
7.415
7.398
7.377
7.357
6.259
6.241
6.213
6.197
5.583
1.605
1.589
13, 500 MHz,
d_6-DMSO
DMSO
H_2O
13
12
11
10
9
8
7
6
5
4
3
2
1
ppm
1.02
1.08
1.07
1.00
2.20
4.08
3.32
2.05
3.28

13, 125 MHz, d_6-DMSO

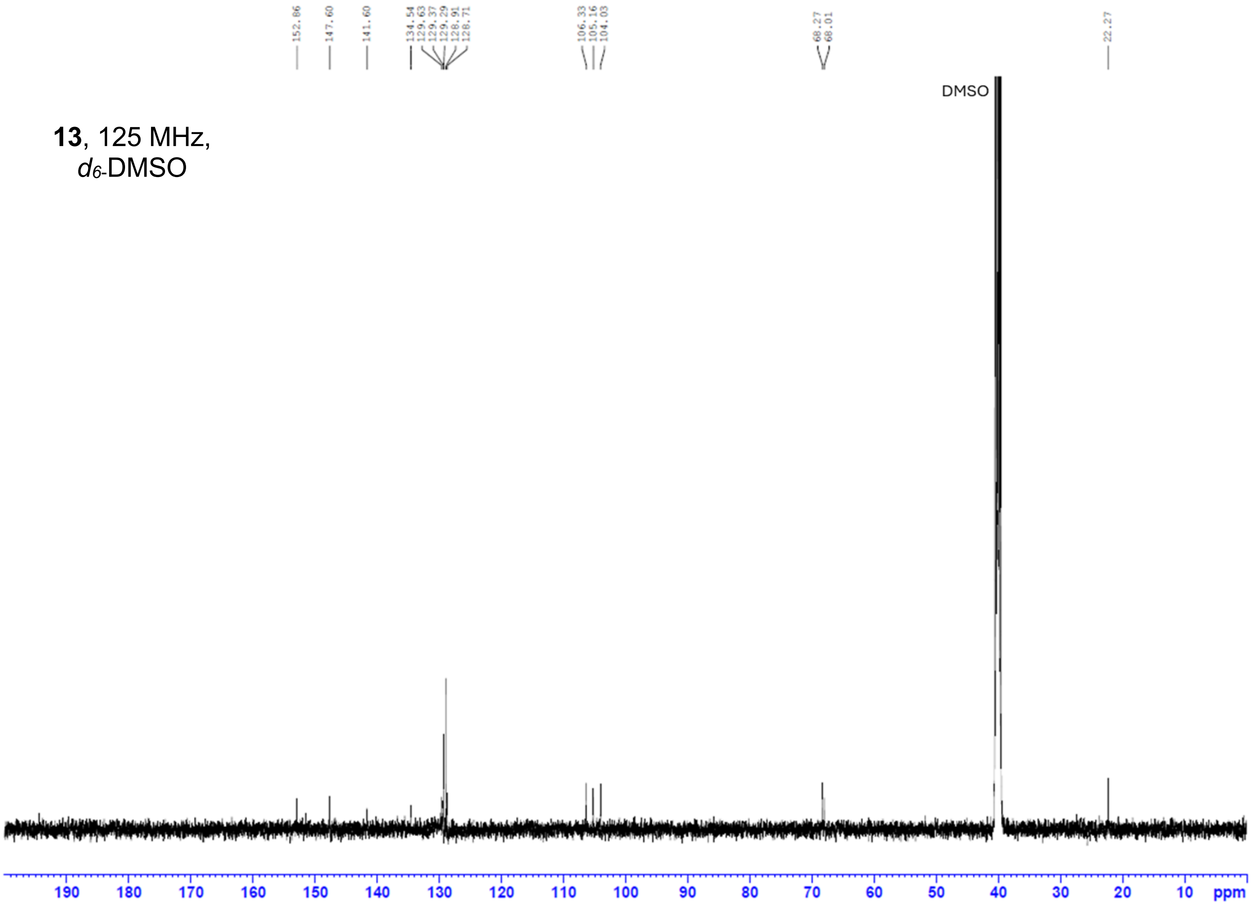

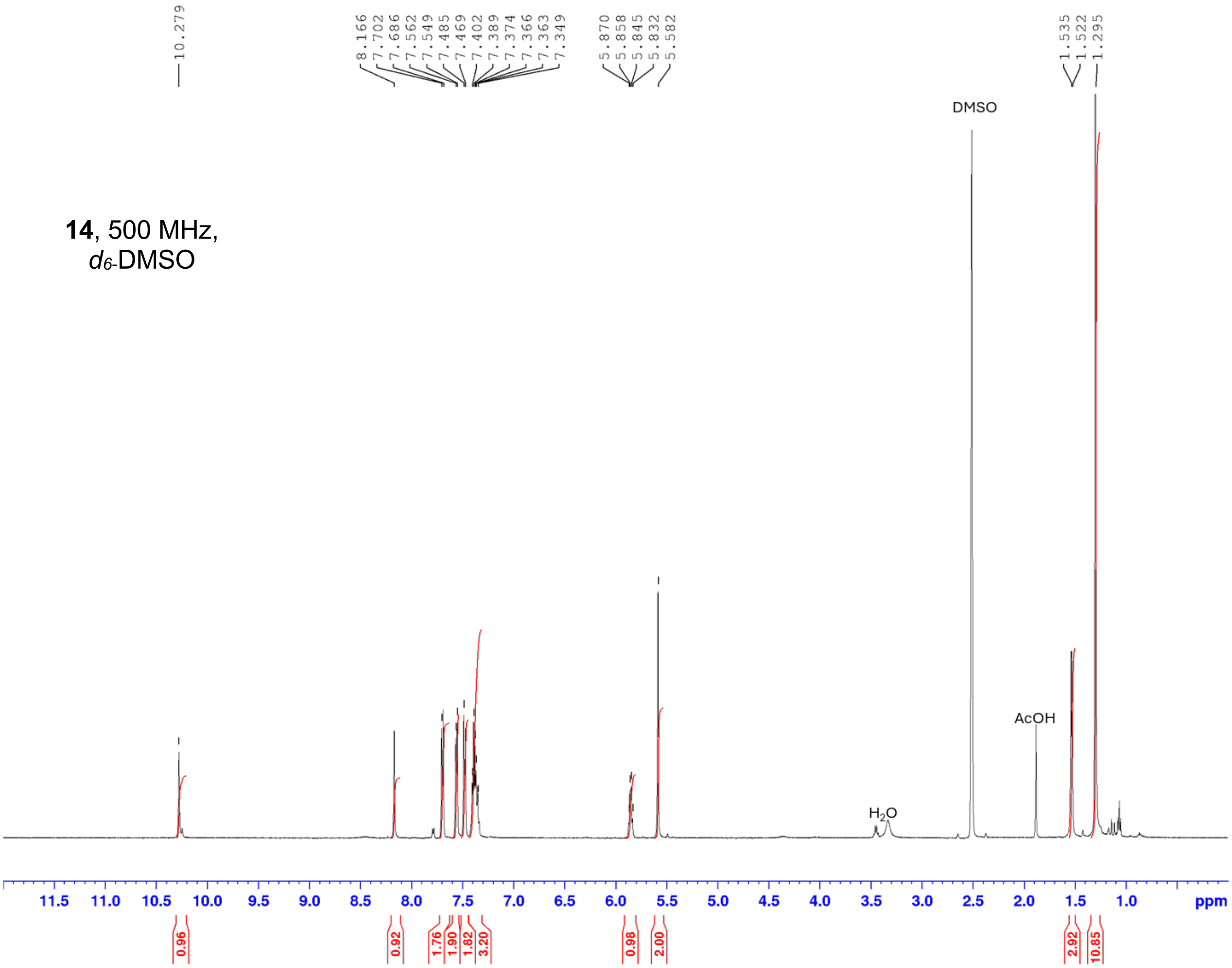

10.279
8.166
7.702
7.686
7.562
7.549
7.485
7.469
7.402
7.389
7.374
7.366
7.363
7.349
5.870
5.858
5.845
5.832
5.582
1.535
1.522
1.295
DMSO
14, 500 MHz,
d6-DMSO
AcOH
H2O
11.5 11.0 10.5 10.0 9.5 9.0 8.5 8.0 7.5 7.0 6.5 6.0 5.5 5.0 4.5 4.0 3.5 3.0 2.5 2.0 1.5 1.0 ppm
0.96
0.92
1.76
1.90
1.82
3.20
0.98
2.00
2.92
10.85

14, 125 MHz, *d*6-DMSO

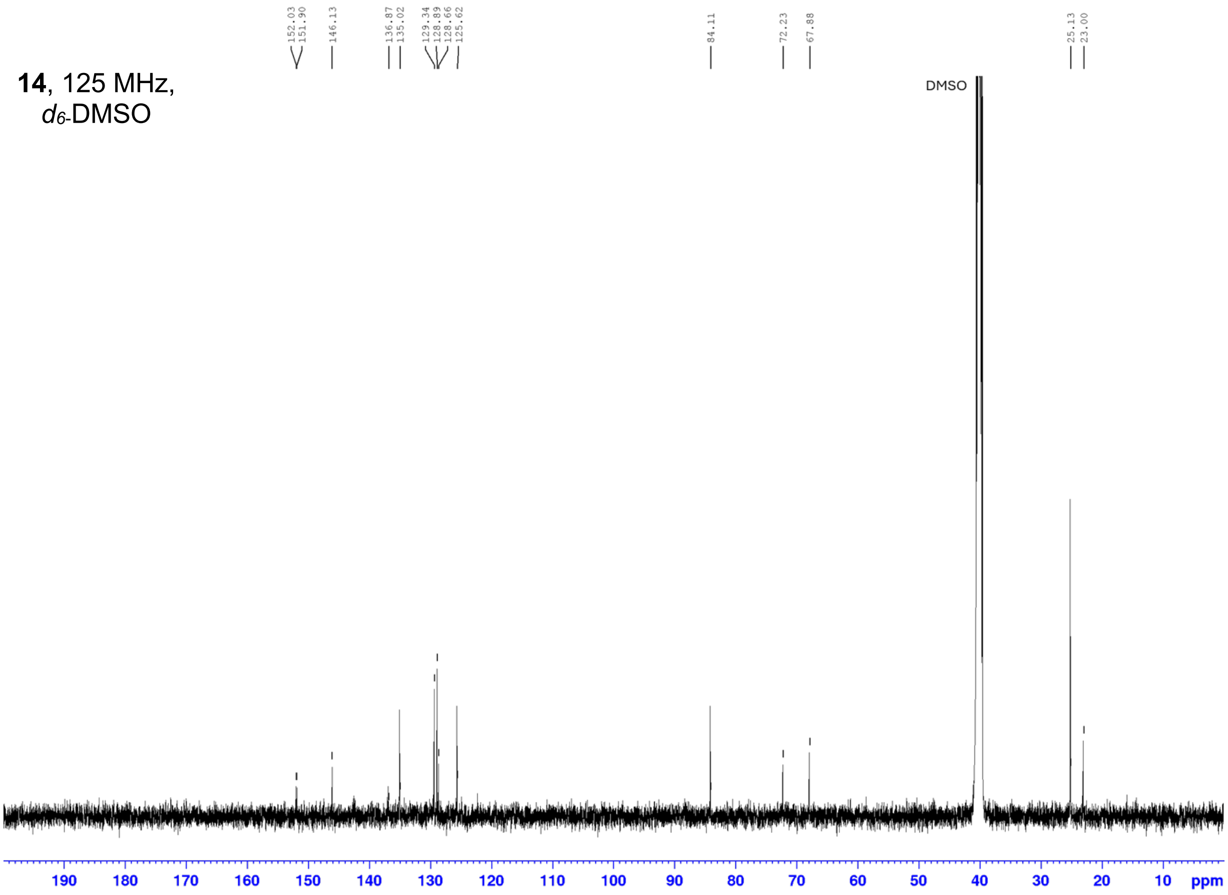

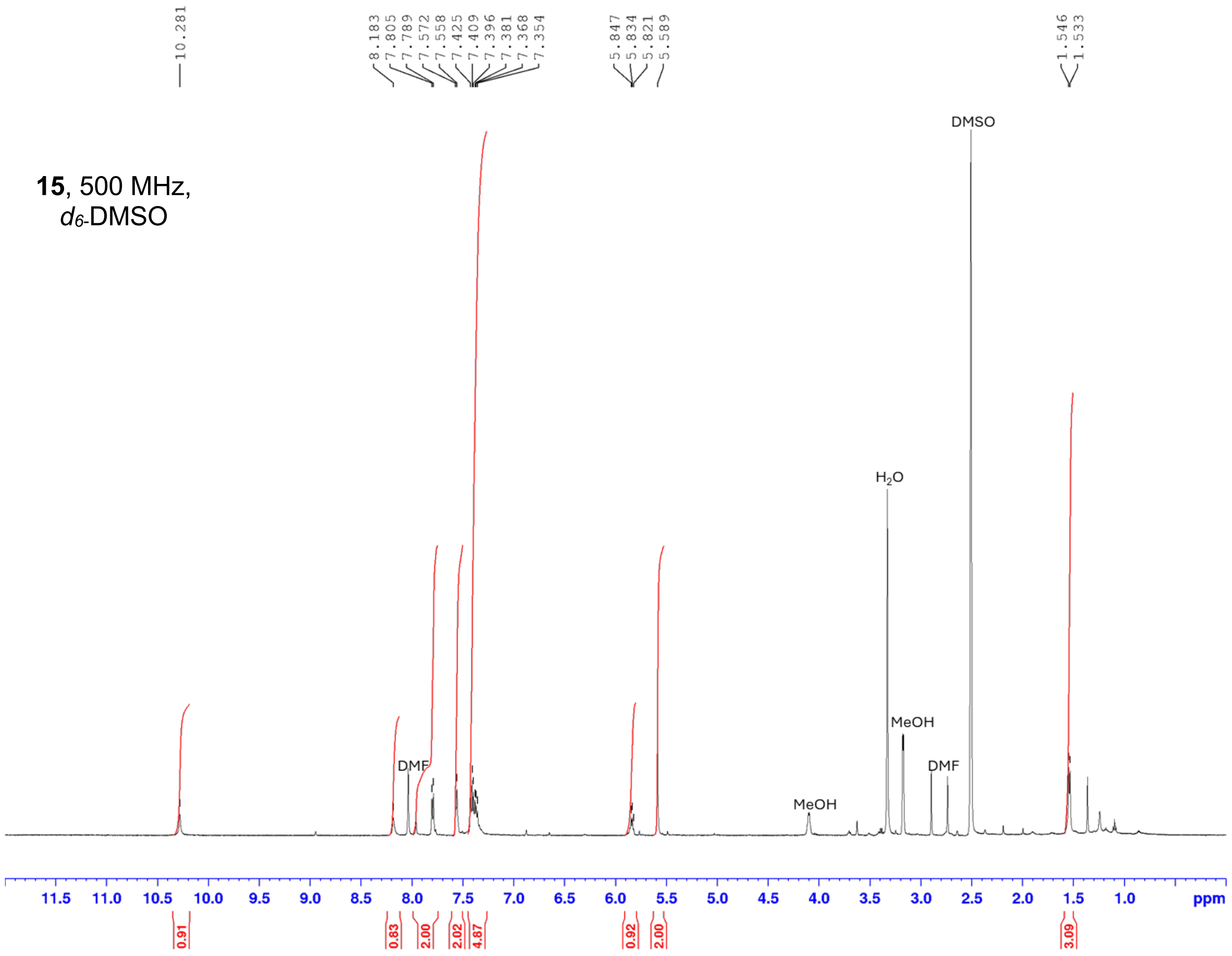

15, 500 MHz,
d6-DMSO
10.281
8.183
7.805
7.789
7.572
7.558
7.425
7.409
7.396
7.381
7.368
7.354
5.847
5.834
5.821
5.589
1.546
1.533
DMSO
H2O
MeOH
DMF
MeOH
DMF
11.5
11.0
10.5
10.0
9.5
9.0
8.5
8.0
7.5
7.0
6.5
6.0
5.5
5.0
4.5
4.0
3.5
3.0
2.5
2.0
1.5
1.0
ppm
0.91
0.83
2.00
2.02
4.87
0.92
2.00
3.09

15, 125 MHz,
d_6-DMSO

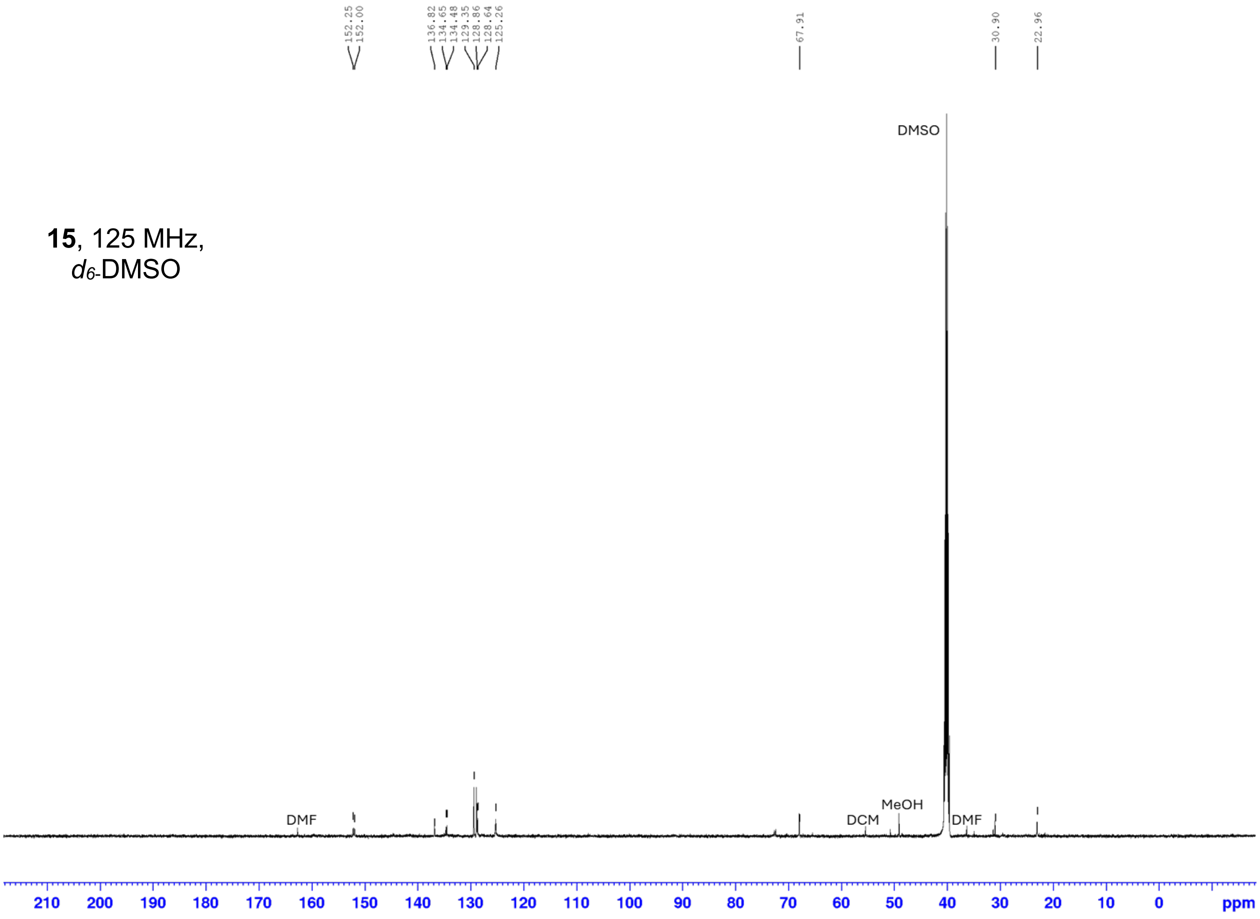

16, 500 MHz, *d_6*-DMSO

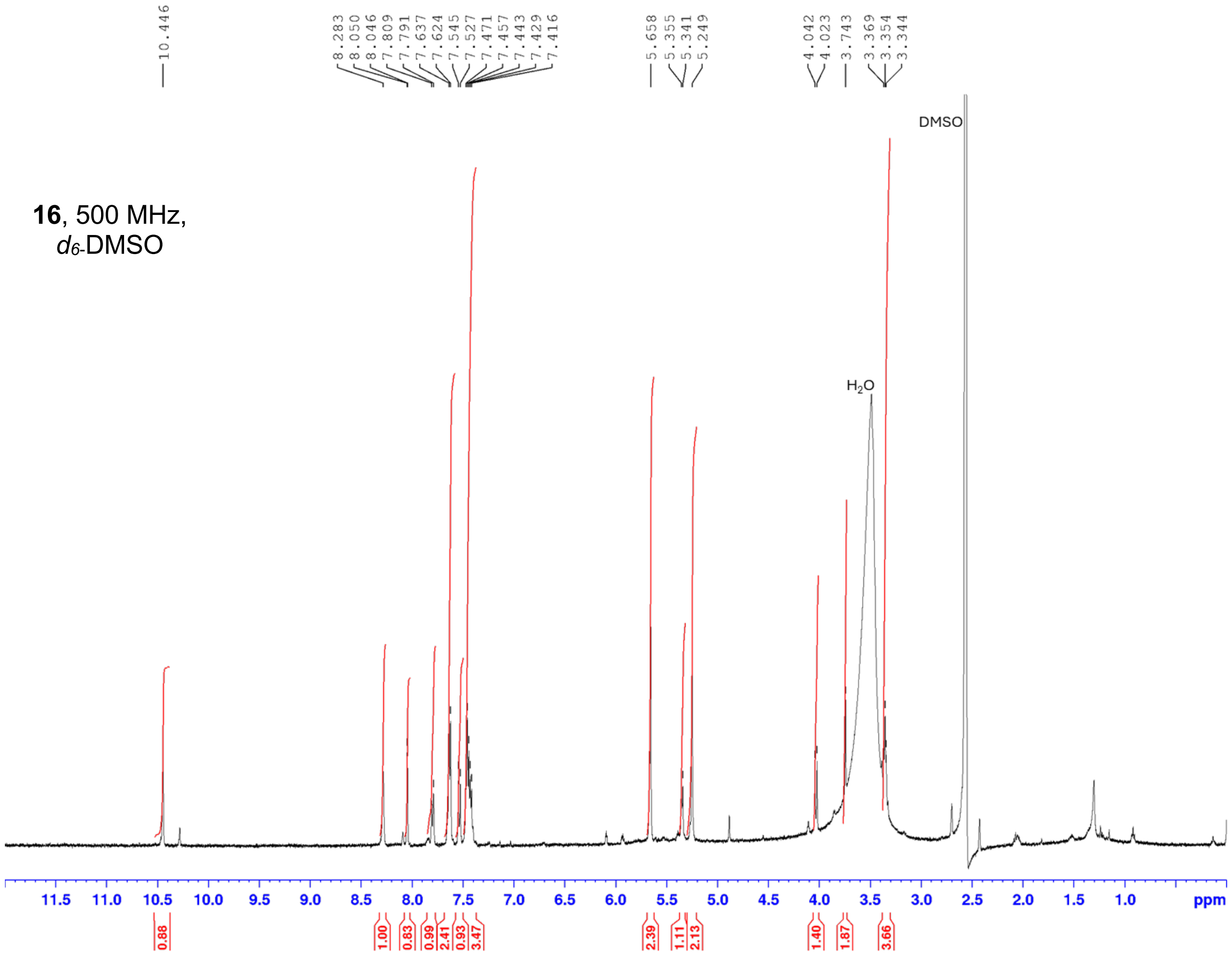

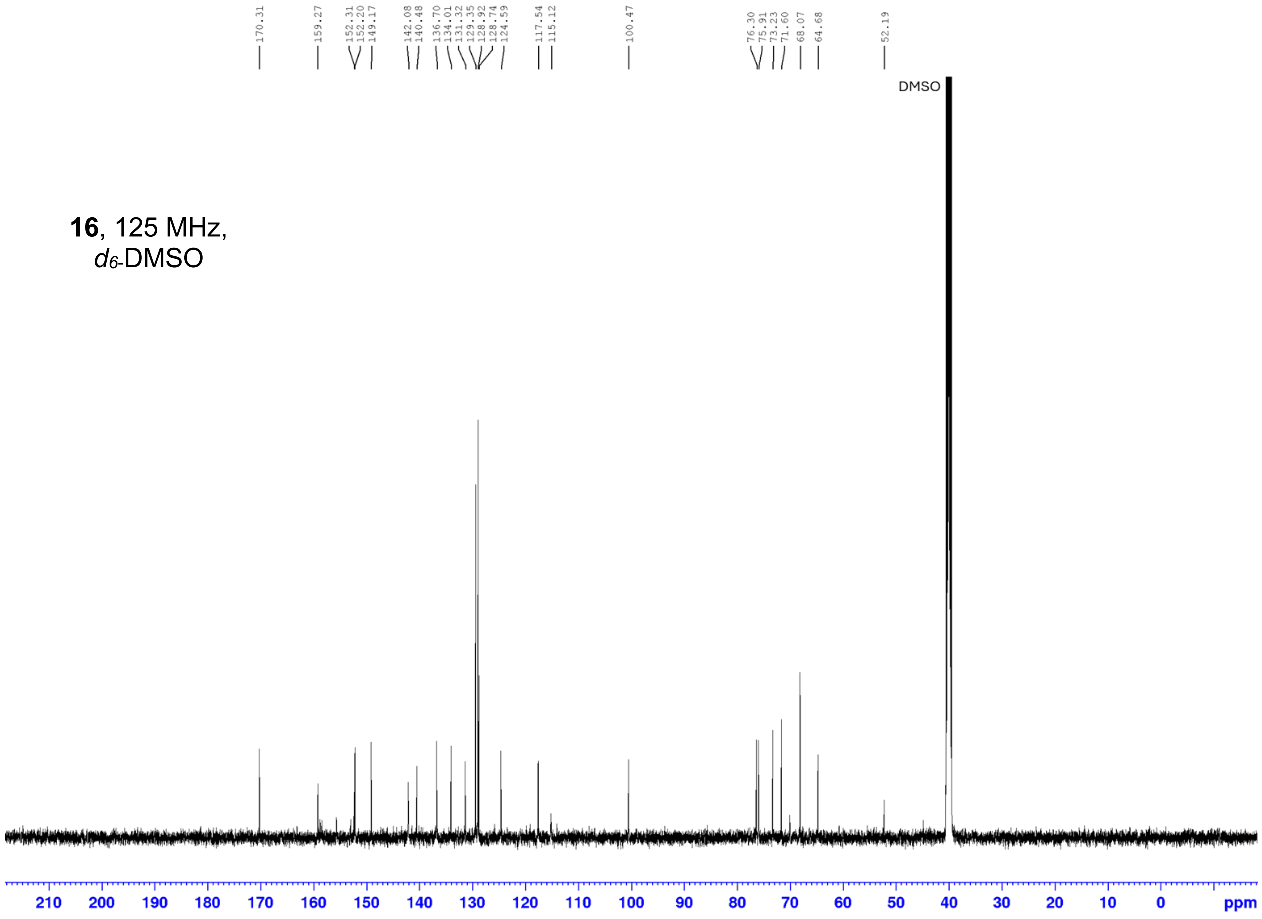
16, 125 MHz,
d6-DMSO
170.31
159.27
152.31
152.20
149.17
142.08
140.48
136.70
134.01
131.32
129.35
128.92
128.74
124.59
117.54
115.12
100.47
76.30
75.91
73.23
71.60
68.07
64.68
52.19
DMSO
210
200
190
180
170
160
150
140
130
120
110
100
90
80
70
60
50
40
30
20
10
0
ppm

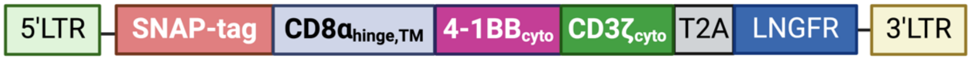

Supporting Figure S1. Diagram of the gene construct used to generate SNAP-CAR T cells with LNGFR expression marker, previously generated in (2). The SNAP-CAR coding region consists of the following components: the SNAP-tag which mediates self-labeling reactions with BG, the CD8α domain hinge and transmembrane domains which positions the SNAP-CAR on the membrane, 4-1BB cytoplasmic domain that provides co-stimulatory signaling, and CD3ζ cytoplasmic domain that provides T cell signaling. The LNGFR protein serves as a CAR transduction marker and is co-expressed via the T2A self-cleaving peptide.

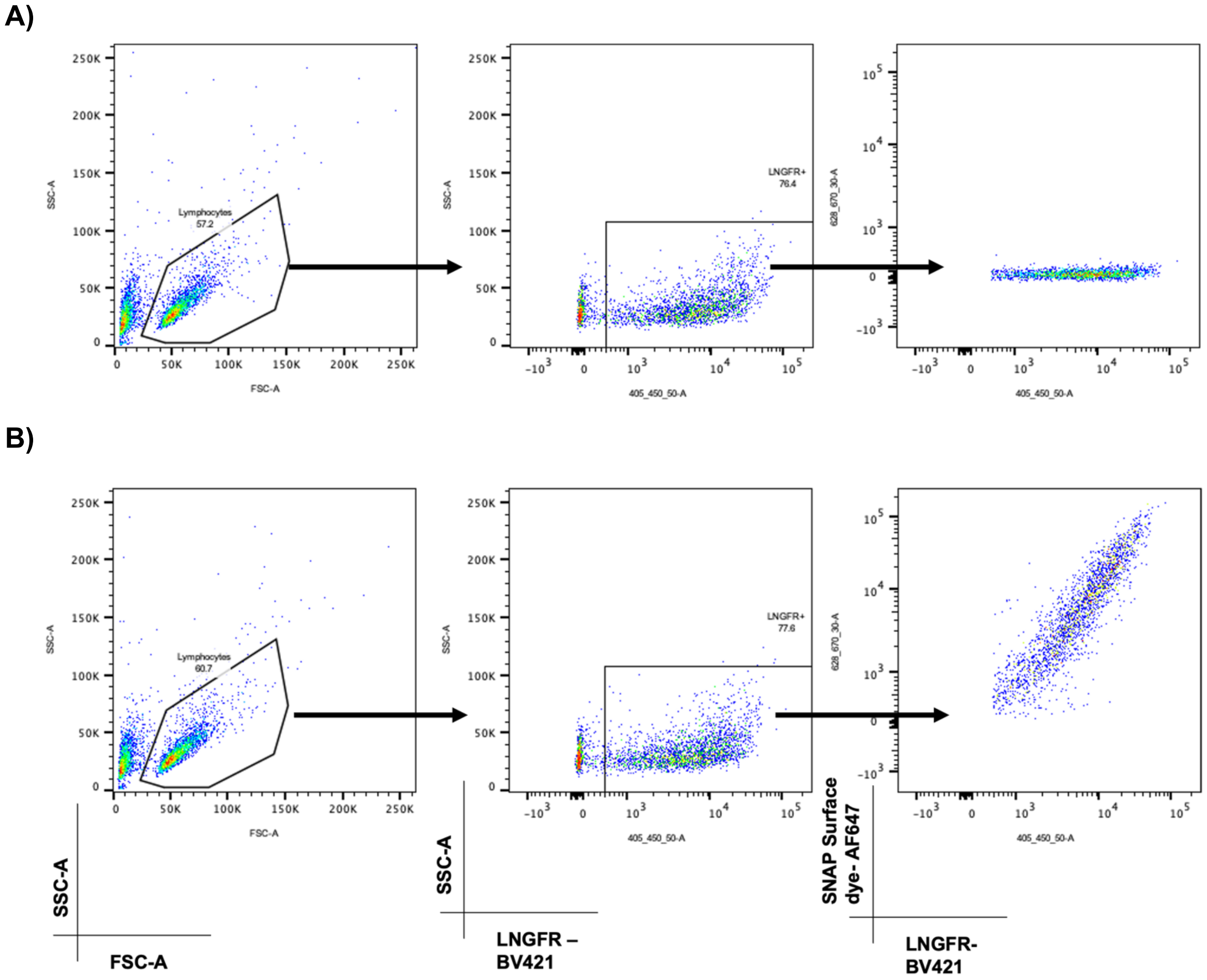

Supporting Figure S2. Representative flow cytometry gating scheme for pulse-chase experiments in Figure 4. Primary human SNAP-CAR T cells were co-incubated with **A**) BG, **1** and no trigger and **B**) TCO-caged BG, **12,** and no dimethyl tetrazine trigger. First live cells were gated based on scatter (Forward Scatter (FSC-A) and Side Scatter (SSC-A)) followed by gating for SNAP-CAR expression (LNGFR-BV421+). Finally, the mean fluorescence intensity (MFI) of SNAP Surface-AF647 dye staining was evaluated for the SNAP-CAR positive (LNGFR-BF421+) cell population. Plots were generated using FlowJo v10.10.0 (FlowJo, LLC).

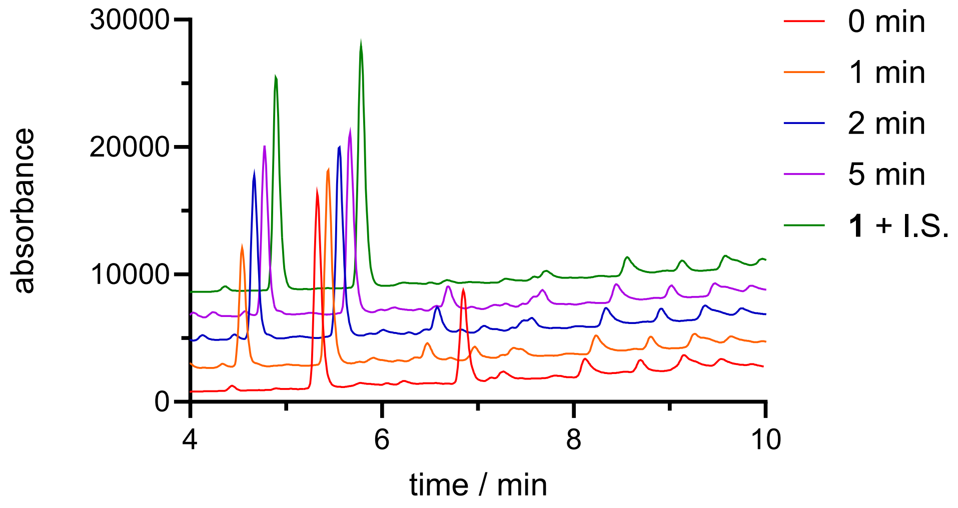

Supporting Figure S3. Representative of an HPLC decaging experiment for Figure 2. Caged compound **13** (20 μM) and 4-nitrobenzyl alcohol (I.S., 20 μM) was dissolved in Tris buffer (4% DMSO) and exposed to 365 nm light for various lengths of time. The samples were analyzed by HPLC using a 10-minute gradient of 25-95% acetonitrile (0.1% TFA) in water (0.1% TFA) and an absorbance of 280 nm. Retention time of **13** is 6.85 minutes, released **1** is 4.49 minutes, and internal standard is 5.38 minutes. Each peak was normalized to the internal standard before analysis.

Supporting References

1. Zhu, R.; Liu, M.-C.; Luo, M.-Z.; Penketh, P. G.; Baumann, R. P.; Shyam, K.; Sartorelli, A. C., 4-Nitrobenzyloxycarbonyl Derivatives of O6-Benzylguanine as Hypoxia-Activated Prodrug Inhibitors of O6-Alkylguanine-DNA Alkyltransferase (AGT), Which Produces Resistance to Agents Targeting the O-6 Position of DNA Guanine. *J. Med. Chem.* **2011**, *54*, 7720.

2. Ruffo, E., Butchy, A.A., Tivon, Y. *et al.* Post-translational Covalent Assembly of CAR and synNotch Receptors for Programmable Antigen Targeting. *Nat. Commun.* **2023**, *14*, 2463.
